# Domains, Feasibility, Effectiveness, Cost, and Acceptability of Telehealth in Aging Care: Scoping Review of Systematic Reviews

**DOI:** 10.2196/40460

**Published:** 2023-04-18

**Authors:** Yichi Zhang, Jessie Siew-Pin Leuk, Wei-Peng Teo

**Affiliations:** 1 Physical Education and Sports Science Academic Group National Institute of Education Nanyang Technological University Singapore Singapore; 2 Ageing Research Institute for Society and Education Interdisciplinary Graduate Programme Nanyang Technological University Singapore Singapore

**Keywords:** telehealth, telemedicine, telecare, telemonitoring, aging care, health care access, scoping review, digital health, mobile health, mHealth, eHealth

## Abstract

**Background:**

Aging is becoming a major global challenge. Compared with younger adults, the older population has greater health needs but faces inadequate access to appropriate, affordable, and high-quality health care. Telehealth can remove geographic and time boundaries, as well as enabling socially isolated and physically homebound people to access a wider range of care options. The impacts of different telehealth interventions in terms of their effectiveness, cost, and acceptability in aging care are still unclear.

**Objective:**

This scoping review of systematic reviews aimed to provide an overview of the domains of telehealth implemented in aging care; synthesize evidence of telehealth’s feasibility, effectiveness, cost benefits, and acceptability in the context of aging care; identify gaps in the literature; and determine the priorities for future research.

**Methods:**

Guided by the methodological framework of the Joanna Briggs Institute, we reviewed systematic reviews concerning all types of telehealth interventions involving direct communication between older users and health care providers. In total, 5 major electronic databases, PubMed, Embase (Ovid), Cochrane Library, CINAHL, and PsycINFO (EBSCO), were searched on September 16, 2021, and an updated search was performed on April 28, 2022, across the same databases as well as the first 10 pages of the Google search.

**Results:**

A total of 29 systematic reviews, including 1 post hoc subanalysis of a previously published large Cochrane systematic review with meta-analysis, were included. Telehealth has been adopted in various domains in aging care, such as cardiovascular diseases, mental health, cognitive impairment, prefrailty and frailty, chronic diseases, and oral health, and it seems to be a promising, feasible, effective, cost-effective, and acceptable alternative to usual care in selected domains. However, it should be noted that the generalizability of the results might be limited, and further studies with larger sample sizes, more rigorous designs, adequate reporting, and more consistently defined outcomes and methodologies are needed. The factors affecting telehealth use among older adults have been categorized into individual, interpersonal, technological, system, and policy levels, which could help direct collaborative efforts toward improving the security, accessibility, and affordability of telehealth as well as better prepare the older population for digital inclusion.

**Conclusions:**

Although telehealth remains in its infancy and there is a lack of high-quality studies to rigorously prove the feasibility, effectiveness, cost benefit, and acceptability of telehealth, mounting evidence has indicated that it could play a promising complementary role in the care of the aging population.

## Introduction

### Background

According to the World Health Organization (WHO) statistics, there were 1 billion people aged ≥60 years in 2020, and this number is projected to reach 1.4 billion by 2030 and double to 2.1 billion by 2050 [1]. The shift in population demographics has substantially contributed to the rising demand for and cost of medical care [2], but many older people are still facing inadequate access to appropriate, affordable, and high-quality health care. In a 2010 survey across 32 countries in Africa, Asia, Eastern Europe, Western Europe, and the Caribbean, 63% of the 1265 respondents aged ≥60 years reported that access to health care when required was a challenge [3].

Telehealth, *the delivery and facilitation of health and health-related services including medical care, provider and patient education, health information services, and self-care via telecommunications and digital communication technologies* [4], is one of the many new possibilities that made health care more accessible and has been widely believed to bring various benefits in aging care settings. First, it can expand health services by removing geographic and time boundaries [5] and enabling socially isolated and physically homebound individuals access to a wider range of care options [6]. Second, it can minimize the risk of direct transmission of infectious agents from person to person [7], especially during the recent COVID-19 pandemic [8]. Third, it redefines health care by engaging the patients’ familiar settings, so that both the patients and the health care providers can put greater emphasis on the intervention itself, which in turn results in improved efficiency and quality of care [9]. Furthermore, as hospitals and medical providers are under increasing pressure to provide quality care at lower costs, telehealth has been accepted and successful across a variety of medical specialties and settings [9], such as dentistry [10], psychiatry [11], dermatology [12], and COVID-19 consultation [13].

The impacts of different telehealth interventions in terms of their effectiveness, cost, and acceptability were studied; however, the results were not consistent [14-16]. For instance, a systematic review of reviews by Ekeland et al [14] in 2010 showed that 21 of the 80 included reviews concluded the effectiveness of telemedicine, 18 found incomplete evidence, the remaining 41 reviews found limited and inconsistent evidence, and the costs of these interventions were not well understood. In 2021, Snoswell et al [16] revisited the meta-analyses from 2010 to 2019 and discovered that telehealth across a range of modalities could be clinically equivalent or more effective than usual care in cardiovascular disease, dermatology, endocrinology, neurology, nephrology, obstetrics, ophthalmology, psychiatry and psychology, pulmonary, and multidisciplinary care. In the same year, Goharinejad et al [15] conducted a review of systematic reviews in the field of telemedicine, in which the 191 included reviews covering different telehealth modalities (eg, telemedicine, telerehabilitation, tele-diabetes, telecardiology, home telecare, telepsychiatry, teledermatology, and teleneurology) and outcomes (eg, clinical outcomes, cost-effectiveness, and user satisfactions) revealed inconsistent evidence regarding the effectiveness (101 positive, 22 unclear, and 1 negative), cost benefits (42 positive and 20 unclear), and satisfaction (47 positive and 9 unclear). In view of the lack of synthesized evidence, particularly in aging care, and the increased demand for telehealth services since the COVID-19 pandemic [17], we would like to extend the literature by including the latest evidence and focusing on the applications of telehealth for the older population.

### Objective

A scoping review generally aims to identify and map the evidence available on a certain topic [18-20]. It is an ideal tool for indicating the volume of literature available and provides a general or detailed overview of the topic’s focus [20]; identifying gaps in the research bases; and evaluating future research priorities in a formal, systematic, and transparent manner [21]. Considering the high heterogeneity of telehealth interventions, we sought to conduct this scoping review to identify the domains in which there is evidence for telehealth’s feasibility, effectiveness, cost benefits, and acceptability in the context of aging care; discover gaps in the literature; and determine the priorities for future research.

## Methods

### Review Methodology

This scoping review of systematic reviews was guided by the methodological framework of the Joanna Briggs Institute [22]. The study selection followed the PRISMA (Preferred Reporting Items for Systematic Review and Meta-Analyses) flow diagram, and the reporting and mapping of the body of literature followed the PRISMA-ScR (Preferred Reporting Items for Systematic Review and Meta-Analyses extension for Scoping Reviews) guidelines [23]. The review protocol was registered in the Open Science Framework [24].

### Selection of the Reviews

The eligibility criteria were established a priori [22]. We included different types of systematic reviews (eg, rapid reviews, narrative reviews, integrative reviews, systematic literature reviews, and systematic reviews with meta-analysis) that analyzed telehealth interventions involving older users or subgroup analysis of older users with or without known health conditions, including those residing in hospitals, nursing homes, and their homes. The intervention could be any form or subgroup analysis of telehealth intervention involving direct communication between older adults and health care providers. No restrictions were placed on the date and location of publications for this review. Only full-text reviews in English were included, considering the language proficiency of the reviewers, to ensure the quality of study selection and data extraction.

The systematic reviews were excluded if (1) the population did not consist of older adults or the reviews did not perform a subgroup analysis of older adults; (2) the reviews solely focused on the design or algorithm of telehealth interventions, policies, or experts’ opinions; (3) the reviews included a broader range of digital health or eHealth interventions but did not present a subgroup analysis of telehealth interventions; (4) the language was not in English; or (5) full texts were not accessible.

### Search for Relevant Studies

#### Source of Studies

In total, 5 electronic databases were searched to ensure comprehensiveness: PubMed, Embase (Ovid), Cochrane Library, CINAHL, and PsycINFO (EBSCO). Reference lists of the included systematic reviews were manually searched to identify potentially relevant reviews.

Haddaway et al [25] recommended Google Scholar search to identify gray literature in evidence reviews; however, Google Scholar is an “academic version of Google” [26] and only consists of a “scholarly” subset of the larger Google search index [27]. Therefore, we decided to use Google to identify any new relevant reviews, to ensure the completeness of the search. As Google’s search algorithm considers multiple factors and signals, we followed the procedure by Piasecki et al [28] and logged out of all Google accounts during the search to avoid personalized search results. Although we were unable to locate any more relevant results on the fifth and sixth pages, we continued browsing and stopped on the 10th page to ensure that there were no further relevant results.

#### Search Strategy

The search strategy for this scoping review used a 3-step search strategy. In the initial step, a limited search was undertaken in Embase (Ovid) for relevant systematic reviews, followed by an analysis of the index terms used to describe the articles and the text words contained in the title and abstract of retrieved papers. This step helped us identify two concepts for the search strategy: (1) aging and (2) telehealth. These two concepts and the choice of databases were discussed and agreed upon in consultation with an experienced librarian (YLM) and all team members. In the second step, all identified keywords and index terms were used to develop our final search strategy, which had been consulted with the librarian (YLM) and compared with the published literature to ensure comprehensiveness. As a result of the preliminary search, some of the possible relevant systematic reviews identified did not include the term “review” in their titles or abstracts; therefore, adding the third concept “review” might result in such reviews being excluded. The detailed search strategy and results across all the included databases are provided in Multimedia Appendix 1. Finally, the reference lists of all identified systematic reviews in the included full texts were searched for additional articles.

#### Selection of Studies

The study selection consisted of two levels of screenings: (1) title and abstract screening and (2) full-text screening, and the reasons for all excluded full texts were recorded. In the first level of screening, 2 independent reviewers (YZ and JSPL) first screened the titles and abstracts of a random sample of 10% (620/6198) of the retrieved articles to ensure consistency in the interpretation of the inclusion and exclusion criteria, while discussions were conducted to reach a consensus in case of any discrepancies. Subsequently, they independently screened the remaining articles, and any study with unclear eligibility was conservatively included in the next step of the full-text screening. Only accessible full-text reviews were considered, and all attempts were made to access full-text copies of the selected articles, with the help of the librarian (YLM) or by directly contacting the author via email.

In the second step, 2 reviewers (YZ and JSPL) independently assessed the full-text articles of all selected reviews. When discrepancies in the assessment were encountered, reviewers discussed among themselves, or with a third reviewer (WPT) acting as a mediator, to achieve consensus.

#### Data Charting

YZ extracted the characteristics of the included systematic reviews using a data charting form, which included the following items: article title, country of the authors, publication year, type of review (with a reason for not conducting a meta-analysis, if applicable), review aim, number of articles included, conceptual and operational definitions of the terms related to telehealth, inclusion and exclusion criteria, outcomes with main findings, quality of evidence, limitation of the reviews, and future practice and research recommendations. Data were manually copied and pasted wherever possible to avoid any potential misinterpretation.

## Results

### Search Results

**Figure 1:**
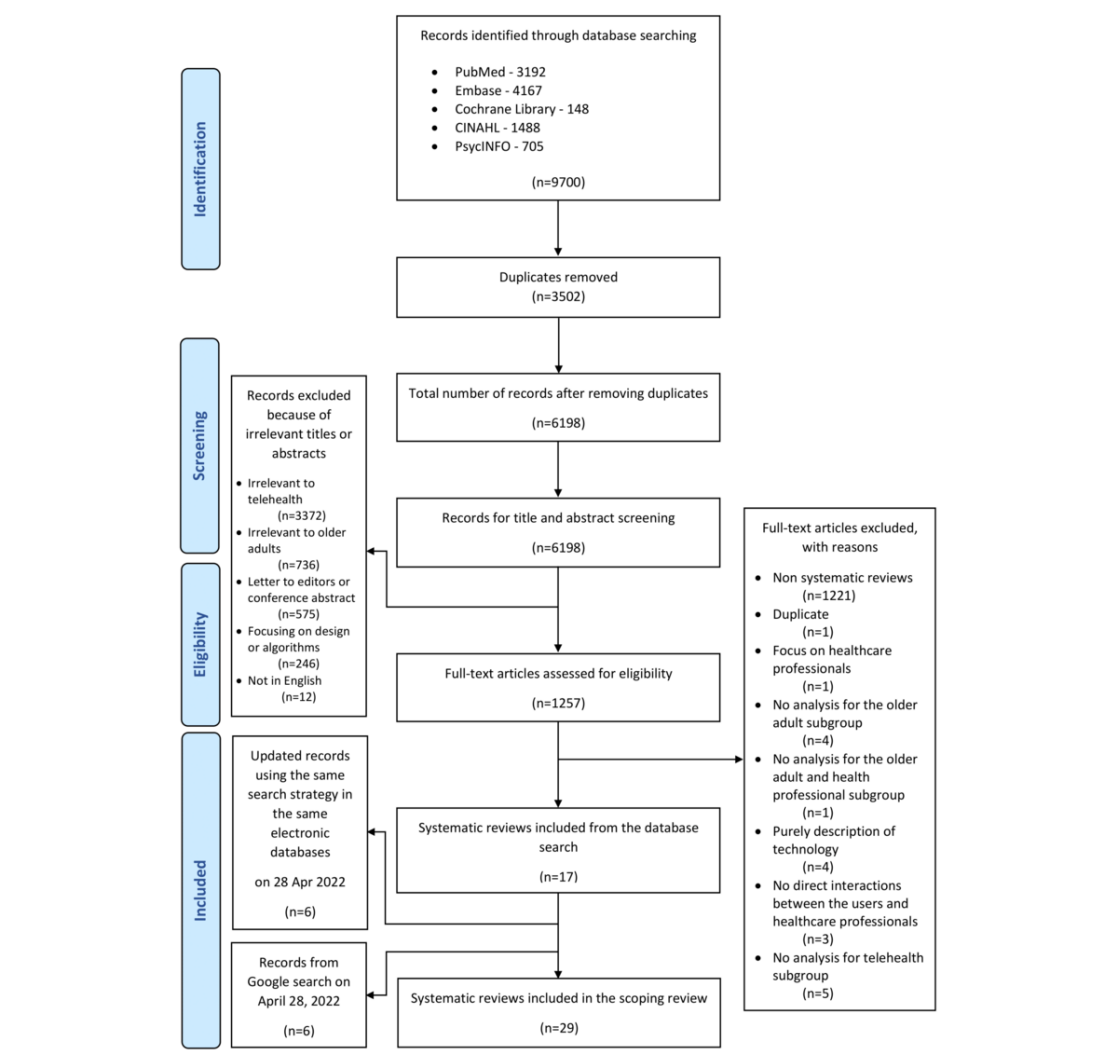
PRISMA (Preferred Reporting Items for Systematic Reviews and Meta-Analyses) 2009 flowchart of the scoping review’s inclusion.

Figure 1 illustrates the preferred reporting items for the PRISMA flowchart of the study selection process. The initial database search conducted on September 16, 2021, identified 9700 articles, and 6198 (63.9%) were included in the title and abstract screening stage after 3502 (36.1%) duplicates were removed. In the full-text screening, 1257 articles were assessed for eligibility, and 17 systematic reviews were found to be relevant, including 1 post hoc subanalysis of a previously published large Cochrane systematic review with meta-analysis [29]. We performed another updated search on April 28, 2022, in the same databases as well as the first 10 pages of the Google search and identified another 12 relevant systematic reviews. As a result, 29 systematic reviews were included for data extraction in this scoping review.

### Characteristics of the Included Systematic Reviews

The characteristics of the included studies are summarized in Multimedia Appendix 2 [30-61].

Of the 29 reviews, 2 (7%) were published before 2010 [62,63], 15 (52%) were published between 2010 and 2019 [29,64-77], and 12 (41%) were published between 2020 and April 2022 [78-89]. A total of 19 reviews provided details on the locations of the included studies [63,68-78,80,81,83,84,86,88,89], with the most prevalent being in North America (the United States and Canada) [63,68-77,80,83,86,88,89], Europe (the United Kingdom, France, Italy, Sweden, the Netherlands, etc) [63,68-71,73-78,80,83,86,88,89], and Australia [68-71,77,78,80,81,86].

The majority of the reviews were narrative, except for 4 that included a meta-analysis [65,74,84,86]. Among the remaining 25 narrative reviews, 5 explained that a meta-analysis was not carried out given the heterogeneity of design, participants, and intervention types [63]; data reporting with the variability of methodology in the studies [67,81]; low quality of the included articles [68]; and high selection and publication bias [78].

A total of 24 reviews presented either a conceptual definition (a working definition in terms of its abstract concept [90,91]) or an operational definition (specific process, events, or activities that the researcher used in the measurement to determine the concept [92]) of at least 1 of the 15 terminologies used (ie, telehealth, telemedicine, telecare, structured telephone, telepsychiatry, remote patient monitoring, teledentistry, telemonitoring, health information technology, remote care programs, telephone only support, remote activity monitoring, telenursing, decision support systems, and health coaching systems), and a summary table can be found in Multimedia Appendix 3.

### Domains of Telehealth Use in Aging Care

#### Population of Interest

Of the 29 included reviews, the population of interest comprised older patients with at least 1 health condition in 17 (59%) reviews [29,62-64,66-69,71-74,78,80,82-84]. Among all these health conditions, the most prevalent ones are cognitive impairment (eg, dementia [82,83] and mild cognitive impairment and Alzheimer disease [66,69]), heart failure [29,80], and prefrailty or frailty [63,74], followed by leg and foot ulcers [72], chronic obstructive pulmonary disease [64], unipolar depression [68], hypertension [84], and oral health [78].

The remaining 12 reviews targeted the general older population, regardless of health conditions. Ten reviews set age cut-offs of 50 [77,79], 55 [87], 60 [70,75,86,89], and 65 years [65,85,88]. Three reviews focused on telehealth services during the COVID-19 pandemic [81,83,88].

It was also reported in several reviews that older adults were not able to participate in telehealth research because of the following factors: sensory change (eg, visual or auditory impairment) [70,82,83], negative affect [83], cognitive impairment [68,70,74,75,82], and communication barriers [70,82].

#### Interventions of Interest

The most prevalent modalities of telehealth interventions in the included reviews focused on remote consultation via mobile phones or video calls [29,64,65,67-69,72,76,77,81], remote monitoring or telemonitoring with synchronous or asynchronous data transmission [64,70-72,85], home-based telecare services [63,66,73,75], and nurse-led telecare services [62,66]. Tam et al [84] examined text messaging in hypertension management; Markert et al [85] investigated remote monitoring combined with health coaching; and the other reviews did not differentiate the different telehealth modalities, with aims including, but not limited to, screening, diagnosis, support, consultation, and education.

According to WHO, universal health coverage is the idea that everyone can access a full range of essential and quality health services, including promotive, preventive, curative, rehabilitative, and palliative care [93]. Most included reviews focused on curative and rehabilitative care (28/29, 97%), and health education interventions were excluded from 3 reviews [29,64,67].

The detailed inclusion (population, intervention, and comparator) and exclusion criteria of the 29 reviews can be found in Multimedia Appendix 4.

### Outcomes With Findings

Outcomes of interest in this review included the effectiveness in individual outcomes (clinical benefits, health literacy, and behavioral outcomes) and system outcomes (efficacy and impact on health system use), feasibility and cost benefits of telehealth interventions or programs, and older people’s acceptance of telehealth with factors affecting their telehealth use.

#### Effectiveness of Telehealth

##### Individual Outcomes: Clinical Benefits

In total, 17 reviews have documented the clinical benefits of telehealth interventions for older adults, in which a clinical benefit is defined as “a favorable effect on a meaningful aspect of how a patient feels (e.g., symptom relief), functions (e.g., improved mobility) or survives as a result of treatment” [94]. In the included reviews, such outcomes included self-reported or clinically assessed health outcomes [62,63,65,66,68-70,72,74,77-79,84,86,87], hospitalization rate [29,81], the mortality rate [29,65], and quality of life [65,77,79,86].

All these reviews have suggested a promising impact of telehealth interventions on the clinical benefits for older adults. Post hoc analysis of a previously published systematic review with meta-analysis by Inglis et al [29] showed that remotely monitoring older patients with heart failure using structured telephone support or telemonitoring could reduce mortality rates and all-cause hospitalization. Likewise, Tam et al [84] performed a meta-analysis to assess the effectiveness of text messaging interventions in hypertension management and concluded that text messaging could substantially reduce systolic blood pressure in older adults. However, there is a lack of rigorous evidence to further support the clinical benefits of these telehealth interventions.

##### Individual Outcomes: Health Literacy

Two reviews reported an improvement in older adults’ health literacy, which is “the achievement of a level of knowledge, personal skills and confidence to take action to improve personal and community health by changing personal lifestyles and living conditions” [95]. Santana et al [66] reported a better understanding of the basic pathology and comorbidities among older adults with Alzheimer disease and an improvement in older adults’ behavior management skills via the use of telecare. Similarly, according to Constanzo et al [69], all included studies concluded that participants with cognitive deficits were able to relearn everyday skills by using different technological tools, particularly when learning methods with error reduction were used.

##### Individual Outcomes: Behavioral Outcomes

The effects of telehealth interventions on various behavioral outcomes of older adults were studied in 3 reviews. van den Berg et al [70] found that regular personal monitoring and individual support by a health care provider or in the form of telemedical measurements seemed to have a positive influence on the adherence to behavioral changes (eg, adherence to medication, diet, physical activity, daily life activities, self-efficacy, and disease management compared with the other outcome categories). Tam et al [84] found a moderate effect on improving medication adherence by integrating telemedicine interventions. Finally, Rush et al [87] presented the use of telehealth as a possible solution to modify older adults’ unhealthy behaviors (eg, smoking) that are higher in rural and remote areas than in urban areas.

##### System Outcomes: Efficacy of the Telehealth Intervention or System

In total, 8 reviews explored the efficacy of telehealth interventions and reached inconsistent conclusions. Jones and Brennan [62], Barlow et al [63], Gentry et al [77], and Markert et al [85] found some evidence of the efficacy of telehealth interventions, although more rigorous evidence is needed. In contrast, Marx et al [65] and Costanzo et al [69] did not find a difference between telemedicine and in-person diagnosis and home visits, and Sekhon et al [82] found inconsistent results on the reliability of telemedicine caused by the testing conditions and the accessibility of telemedicine. In addition, Markert et al [85] reported that the presence of humans in the interventions might influence the outcomes. Jones and Brennan [62] revealed that the use of telehealth for clinical assessment has shown great promise in the nursing process; however, it was not ready for wide-scale clinical deployment.

##### System Outcomes: Impact of Telehealth on the Health Care System Infrastructure

In total, 7 reviews included the impact of telehealth on health care system use as an outcome. Barlow et al [63] showed that telephone follow-ups after hospital discharge were associated with reduced health service use (eg, lower hospital admissions and costs), Franek [64] reported that home telemonitoring could reduce the use of other health care services with a need of further confirmation with more randomized controlled trials (RCTs) of high quality, and Murphy et al [81] demonstrated that a telemedicine-based geriatric clinic model of care had the potential to reduce acute hospitalization and shorten the waiting times. The other 4 reviews demonstrated the potential benefits of telehealth services in improving older adults’ access to health care [68,83,88] and extending existing health services from the health care facilities to home and community [86].

A summary of the effectiveness outcomes and findings can be found in Multimedia Appendix 5.

#### Feasibility of Telehealth

To measure the feasibility of telehealth, earlier studies used different indicators, such as use [96], adherence [97], dropout rates [98], technical errors [98], specialist consultation time [98], perceived feasibility [99], delivery mode [100], and social accountability [100]. Considering the lack of details in the included reviews, we opted to use adherence and attrition rates as proxy measures.

Three reviews reported on older users’ adherence to telehealth interventions. In the review by Sekhon et al [82], mixed results were reported on the actual rate of adherence to telehealth and the implementation of telemedicine specialists’ recommendations. In addition, Rush et al [87] also indicated that half of the studies reported low adherence and modest attrition rates because of technology failure and not achieving behavioral goals, and the adherence rates were found to vary according to the nature of telehealth interventions. Santana et al [66] reported an improvement in adherence to treatment, an increase in the number of older adults accompanied by health providers, and an improvement in the quality of care. The review with meta-analysis by Marx et al [65] found that more than half of the studies (7/9, 78%) found a much lower attrition rate among those who used telephone consultations (0%-31%) than those who used telemonitoring devices (50%-61%). A summary of the feasibility outcomes and findings can be found in Multimedia Appendix 6.

#### Cost Benefits

In total, 6 reviews examined the cost benefits of the telehealth interventions. Marx et al [65] concluded that telehealth interventions were cost-effective compared with no intervention, but the cost-efficacy compared with home visits was not yet established. Peretz et al [73] found no reliable cost estimates for remote patient monitoring program implementation, but it appeared that the cost of remote patient monitoring programs was dependent on the number of vital signs monitored, the complexity of the health condition monitored, and the geographic locations of the programs. Among the studies included in the review by Gentry et al [77], only 1 found no health care cost difference between virtual consultation and in-person treatment, whereas the other studies on memory disorder clinics via telemedicine only supported cost benefits to patients and caregivers but found no evidence of cost-effectiveness for health care organizations. In dentistry, Aquilanti et al [78] found that telehealth interventions tended to be less costly than face-to-face oral examinations. In the review by Murphy et al [81], virtual geriatric clinics were likely to be more cost-effective, but substantial discrepancies were noted in 2 studies because they used different costing models. The review by Rush et al [87] included studies of medium to high quality and observed direct cost savings for the health care system and rural older adults; however, many of the cost savings resulted from the savings on travel expenses. A summary of the cost-benefit outcomes and findings can be found in Multimedia Appendix 7.

#### Acceptance of Telehealth

A summary of the acceptance outcomes and findings can be found in Multimedia Appendix 8.

##### Satisfaction, Acceptability, Attitude, Experience, and Usability

In total, 12 reviews reported users’ attitudes, satisfaction, or acceptance of telehealth services. Narasimha et al [76] reported that 65% of the geriatric population has shown a strong will to keep abreast of current advances, despite the stereotype that older people may be more averse to using technology for health care. Positive attitude toward telehealth was also reported in other reviews [62,66-68,77,78,81-83]. If the attrition rate could be used as a proxy for older users’ acceptance, Marx et al [65] found that among the geriatric population, acceptability was good for telehealth consultation but less desirable for asynchronous approaches that relied on computerized devices. Moreover, Costanzo et al [69] discovered that younger caregivers seemed more comfortable and capable of using the internet and were more motivated to use the service.

##### Factors Affecting Older Adults’ Use of Telehealth: Overview

**Figure 2:**
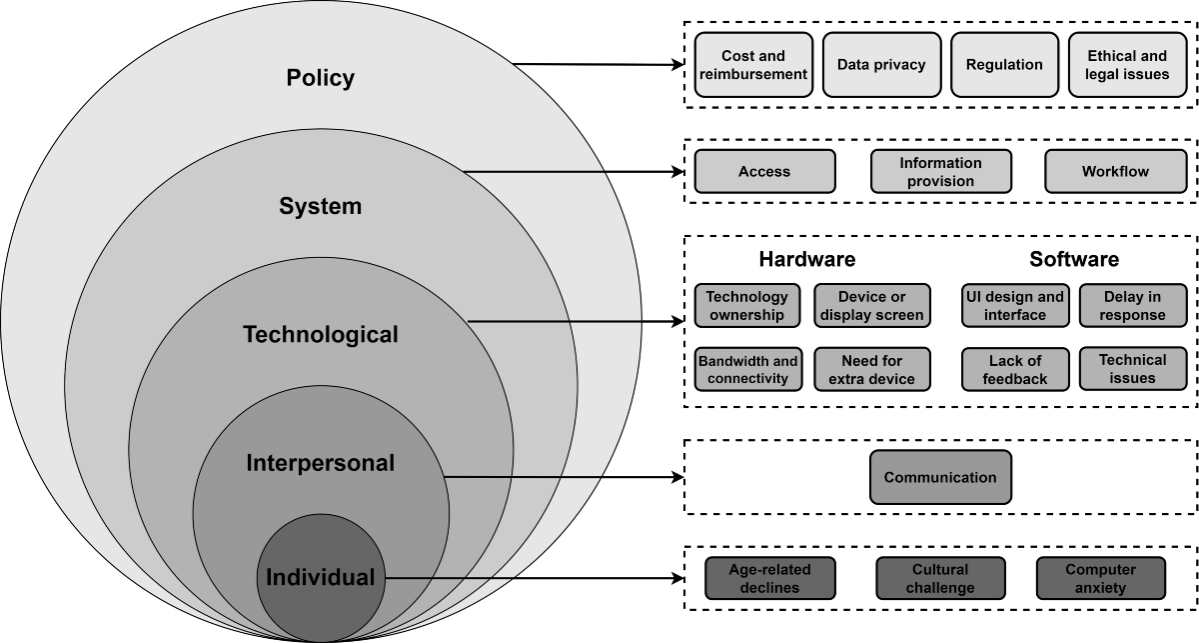
Illustration of our model, which was adapted from the social ecological model [101]. UI: user interface.

In the 1970s, Urie Bronfenbrenner developed the social ecological model as a conceptual model to understand human development. It consists of nested circles centered at individuals and contains microsystem, mesosystem, exosystem, macrosystem, and chronosystem levels [101]. In this review, we adapted the social ecological model as a framework to guide the classification of the factors affecting older adults’ telehealth use, namely, individual, interpersonal, technological, system, and policy levels (Figure 2 [101]).

##### Factors Affecting Older Adults’ Use of Telehealth: Individual Level

At the individual level, aging-related declines in vision, perception, hearing, motor, and cognitive functions adversely affected older adults’ ability to carry out tasks, thus increasing the challenge of telehealth use and the inaccuracy of assessment [67,68,76,77,79]. For older adults who are unfamiliar with technologies, telehealth may bring technical difficulties and cultural challenges [71,83], and some even dropped out of the studies [70]. Such factors may lead to resistance to technology use [68].

##### Factors Affecting Older Adults’ Use of Telehealth: Interpersonal Level

Some health care providers reported difficulty in communicating and conducting proper physical examinations via telehealth owing to older patients’ possible age-related declines and technological incapability [67,68,83]; therefore, they were reluctant to recommend technologies. Moreover, it was recommended that more communication between patients and staff as well as between peers could create a feeling of more involved care [80].

##### Factors Affecting Older Adults’ Use of Telehealth: Technological Level

At the technological level, both hardware and software factors have been reported to affect telehealth use in the older population.

Regarding hardware factors, ownership of technology [79], an effective device or display screen [71,76], bandwidth, and connectivity [77,83], along with a need for devices with widgets or multiple screens [71], could influence older users’ ability to accomplish the final goal. For example, some patients with hearing disabilities reported interference between a videophone and their hearing aids [76]. The convenience of technology use can improve comfort and efficiency.

Regarding software factors, several reviews identified barriers in terms of the software and user interface design, such as inappropriate font size, unusual characters, bland graphics, poor color contrast, and complicated menu designs [76,79]. A simple and intuitive interface that requires little or no technical knowledge would better reflect normal daily activities and allow a more seamless transition toward its use [80], whereas a delay in responses, lack of feedback, and technical issues may lead to frustration for older users, which may lower their motivation to continue telehealth use [71].

##### Factors Affecting Older Adults’ Use of Telehealth: System Level

An important factor regarding older adults’ telehealth use is access, which may be limited by age-related sensory impairment [67,68] and unfamiliarity with technology [68], as reported at the interpersonal level. Sekhon et al [82] also reported that gaining a referral to a specialist who uses telemedicine was another barrier, although all physicians claimed future use of telehealth. In addition, skepticism about the telehealth benefits [68] and information loss owing to the inability to properly examine patients [83] were other 2 barriers reported by health care providers.

##### Factors Affecting Older Adults’ Use of Telehealth: Policy Level

At the policy level, several reviews reported that the cost of using telehealth and reimbursement from the government or insurance companies were barriers to telehealth adoption [77,79]. Other barriers reported at the policy level are regulations (eg, state law and licensure) as well as ethical and legal issues (liability, malpractice, and safety) [77,89].

Data privacy was a contradictory factor in these reviews. In the review by Karlsen et al [75], possible privacy issues caused by the use of cameras and video recording tools were not seen as a problem by most older adults because the technologies were supposed to help them live safely in their own homes. In contrast, the reviews by Pool et al [89] and Kruse et al [79] reported that the privacy issue had an impact on user attitudes, intentions to adopt, and their actual use of telehealth.

### Quality of the Evidence Included in the Reviews and Limitations of the Reviews

Most of the included reviews reported that the literature did not meet orthodox quality standards because of the study design in the lower tiers of the hierarchy of evidence [63,69,71,72,79,81]; small sample size [63,65,68,69,71,82,86]; short follow-up period [63]; small number of studies [63,65,66,68,79,81,84]; high risk of bias [67,71,72,74,84]; high heterogeneity of interventions and outcomes [64,67,69,78,81,86,87]; insufficient and partly inadequate reporting of predefined outcome values and few participants, especially in the intervention group [72]; and inconsistent measurement of the outcomes [67,69,87]. The included studies of all the reviews were conducted in a limited number of locations (Multimedia Appendix 2), and the interventions used different telehealth modalities in different settings; hence, generalizability was attenuated [64,68,75,81], and the findings might not be generalizable to the entire older population group.

## Discussion

### Principal Findings

This scoping review synthesized the evidence from past research on telehealth in aging care and summarized the findings of 29 systematic reviews regarding telehealth interventions in aging care. Although telehealth has garnered attention since before 2010, it was in the spotlight during the COVID-19 pandemic when >40% of the included reviews were published. The present evidence shows promising evidence regarding the feasibility, costs, and acceptability of telehealth for screening, diagnosing, supporting, and consulting in aging care. However, some discrepancies were observed because of differences in telehealth modalities, health conditions, definitions of outcomes and measurements, and costing models. In addition, we summarized and categorized various factors affecting older people’s telehealth use into individual, interpersonal, technological, system, and policy levels to provide a pathway for collaborative efforts to better prepare the older population for digital inclusion.

A large proportion of the evidence focused on curative and rehabilitative care (28/29, 97%), and there is scarce evidence on promotive, preventive, and palliative care, which is consistent with the scoping review in 2013 that the largest number of studies primarily focused on chronic disease management and symptom management [102]. Despite the importance of health education and promotion for the whole population, older adults have long been left out of health promotion activities until after 2001 when WHO experts declared that a healthy lifestyle should be emphasized for all ages [103]. Evidence has indicated that a healthy lifestyle, such as quitting smoking, limiting alcohol consumption, and increasing physical exercise, can help delay the development of many diseases, prevent the loss of functional capacity, improve the quality of life, and extend life expectancy [103]. Palliative care is another important public health issue as a consequence of the aging population, which focuses on improving the quality of life and dignity of people facing the end of their lives as well as the support and care of their loved ones [104]. For most patients in need of palliative care, the most preferred place of care is at home [105]. Nevertheless, some unmet in-home palliative care needs, such as the lack of communication among health care providers and patients’ uncertainty about the urgency of their problem, have also been reported [106]. Future research could investigate how telehealth can address these gaps and the role of telehealth in universal health coverage.

In comparison with usual care, different modalities of telehealth have demonstrated remarkably promising effectiveness in improving both individual outcomes and system outcomes. As Russell et al [107] stressed that it is important to ensure that telehealth is not inferior to usual care, our results support past reviews [14-16] that telehealth could be a viable alternative to traditional clinical practice in selected domains and further highlight that telehealth could be effectively used in a broad range of clinical disciplines. However, we should be cautious about possible biases in the literature that may limit the generalizability of the results. For instance, most empirical studies in the included reviews were conducted in countries with higher incomes, and older adults with chronic or aging-related conditions were excluded in some studies if they were not part of the target population. Moreover, our findings also reiterate the inconsistent quality of evidence reported in other reviews [14,108]. The included reviews were predominantly narrative (25/29, 86%), and most reported that the literature was not up to orthodox standards. The evidence base needs to be strengthened through additional studies on the top tiers of the hierarchy of evidence (eg, RCTs or cluster RCTs) with larger sample sizes, longer follow-up periods, and consistent definitions and outcome measures coupled with good reporting methodologies.

In terms of the feasibility of telehealth in aging care, we used users’ adherence and attrition as proxy measures and found mixed results, varying by different telehealth modalities and subpopulation types. Indeed, suboptimal adherence and substantial attrition are common in digital health intervention studies among the older population [109,110], and some contributing factors include personal choices, technical difficulties, physical and cognitive impairments, and concerns regarding the security of digital health interventions [110]. Low engagement continues to plague the internet-based studies [111], results in a study cohort not being representative of the demographics and disease status of the originally recruited study population [109], thereby threatening the validity of the findings [112]. Meanwhile, other factors such as referral by a clinician to the study, compensation for participation, having a clinical condition of interest in the study, and an older age [109] have been revealed to be associated with increased participation retention. Future research could further explore how older users’ adherence and retention rates could be improved to advance the current telehealth practices and how this could impact the effectiveness, costs, and acceptability of telehealth interventions.

Similar to the systematic review of reviews by Ekeland et al [14], several reviews in our study also reported promising benefits of telehealth in terms of cost, and we also observed some discrepancies in implementation cost savings and cost-effectiveness in different studies owing to heterogeneity in comparator care delivery modes [65], the complexity of health conditions [73], geographic locations [73], and cost models used [77,81]. A common obstacle in most published economic evaluations of digital health interventions is reliance on standard methodological recommendations for assessing health care technologies, but such methodological assumptions may not fully reflect the nature of digital health interventions, especially complex ones [113]. Moreover, the cost benefits of digital health interventions may vary over time as well as the degree to which users use them, making their impact more likely to be heterogeneous [113-115]. Further research could overcome these challenges by streamlining the methodologies. For example, as recommended by Gomes et al [113], researchers should carefully choose comparators, determine the scope of cost and effects to be considered, and identify the effects of the interventions with appropriate measurements as well as the cost of other resources before the economic analysis.

In contrast to other studies indicating that older adults have not been fully ready for telehealth [116,117], all our included reviews that assessed the participants’ experiences with and attitudes toward telehealth interventions have demonstrated good acceptance of telehealth among the older population. This difference might be explained by the use of different telehealth modalities in different studies as well as the prescreening process in some studies that excluded older adults who may have difficulty using telehealth. We identified a similar set of factors affecting older adults’ telehealth use as other reviews [118,119], and further categorizing these factors into individual, interpersonal, technological, system, and policy levels could assist in understanding the needs of the older population in this process and identify the potential collaborative efforts that individuals, health care providers, developers of telehealth applications, government and community organizations, and policy makers can make to prepare the older population for digital inclusion. To help older individuals cope with the cultural and psychological challenges associated with digital health technologies, training programs could be offered to both the older population and health care providers to improve their digital literacy as well as skills in interpersonal communication and rapport building. Although there is no “one-size-fits-all” solution, developers of telehealth applications could engage older people, especially those with special needs (eg, those with physical immobility, sensory change, negative affect, cognitive impairment, low digital literacy, or communication barriers), in the user-centered design, testing, and evaluation of telehealth technologies and keep their needs in mind. Health systems should be responsible for ensuring a smooth and continuous clinical workflow with telehealth, along with providing the latest guidelines and health information to health care providers and older patients. Policy makers could enhance clinical guidelines and policies to regulate the design and implementation of telehealth, address privacy concerns, bridge the digital divide, and improve the current payment models so that telehealth could be offered to users with better security, accessibility, and affordability.

### Strengths and Limitations

We chose to conduct a scoping review to comprehensively cover a wide range of older subpopulations, telehealth interventions, outcome measures, and types of systematic reviews. Our choice of only including systematic reviews was in response to the rapid growth of telehealth studies and the heterogeneous aims of the systematic reviews of telehealth interventions in the aging care context. Nonetheless, this choice may have neglected the details of the included telehealth interventions as well as the exclusion of some pertinent studies in consideration of their methodology and design. Although we adopted a systematic approach guided by the methodological framework of the Joanna Briggs Institute [22], there might be undetected relevant systematic reviews. The inclusion of only full-text systematic reviews in English may also lead to the loss of sight of those reviews without full-text accessibility. In addition, the low number of systematic reviews with meta-analyses also limits the robustness of the conclusions that can be drawn.

In addition to the methodological limitations, we identified limitations regarding the generalizability of the results. First, most of the studies in the included reviews were conducted in North America, Europe, and Australia, which may limit the generalizability of our findings to the global telehealth market. Second, several reviews have reported that the prescreening process has excluded the older users with special needs (eg, physical immobility, sensory change, negative affect, cognitive impairment, and communication barriers); hence, the results may not be generalizable to these vulnerable groups. Third, we identified different definitions of the population (older adults), interventions (telehealth, telemedicine, and telecare), and outcome measures, which might be because of different search terms, research settings, and geographic locations. This makes it difficult to compare the feasibility, effectiveness, cost, and acceptability of the interventions. Therefore, it is important to standardize and carefully define the terminologies and assessment tools in future research to reduce bias and draw robust and reliable conclusions.

### Conclusions

The development and implementation of telehealth have been further catalyzed by the COVID-19 pandemic, and this scoping review has identified considerable evidence of the effectiveness, feasibility, cost benefits, and acceptability of telehealth applications in aging care. Although telehealth remains in its infancy and there is a lack of high-quality studies to draw robust conclusions, mounting evidence indicates that telehealth plays an important complementary role in the care of the aging population. It is imperative for older individuals, health care providers, developers of telehealth applications, government and community organizations, and policy makers to make a collaborative effort. This could help gain deeper insights into the multifaceted needs of and challenges faced by the older populations; facilitate a user-centered approach in the design and testing of telehealth technologies; and improve the security, accessibility, and affordability by enhancing the existing clinical guidelines and regulations. More high-quality studies are also required to provide a robust evidence base for aging care.

## Appendix

Multimedia Appendix 1 Search strategy and results.

Multimedia Appendix 2 Characteristics of the included reviews.

Multimedia Appendix 3 Summary of the conceptual and operational definitions of the terminologies.

Multimedia Appendix 4 Inclusion and exclusion criteria of the included reviews.

Multimedia Appendix 5 Summary of the effectiveness outcomes of telehealth.

Multimedia Appendix 6 Summary of the feasibility outcomes of telehealth.

Multimedia Appendix 7 Summary of the cost-benefit outcomes of telehealth.

Multimedia Appendix 8 Summary of the acceptance outcomes with factors affecting telehealth use.

## Abbreviations

PRISMA: Preferred Reporting Items for Systematic Review and Meta-Analyses
PRISMA-ScR: Preferred Reporting Items for Systematic Review and Meta-Analyses extension for Scoping Reviews
RCT: randomized controlled trial
WHO: World Health Organization
