# Supplementary material for: Domains, Feasibility, Effectiveness, Cost, and Acceptability of Telehealth in Aging Care: Scoping Review of Systematic Reviews
Source: JMIR Aging. 2023 Apr 18;6:e40460. doi: 10.2196/40460 (PMC10155091; doi:10.2196/40460)
Supplement: Multimedia Appendix 1 [file aging_v6i1e40460_app1.docx]

Multimedia Appendix 1. Search strategy and results

Search strategy and result for Embase (Ovid) <1974 to 2021 September 14> (searched on 2021 Sep 16th)

| **Search number** | **Query** | **Results** |
| --- | --- | --- |
| 6 | limit 5 to english language | 4,167 |
| 5 | 1 and 4 | 4,308 |
| 4 | 2 or 3 | 100,234 |
| 3 | (telehealth or tele-health or "tele health" or telemedicine or tele-medicine or "tele medicine" or "mobile health" or mHealth or "m health" or m-health or "electronic health" or eHealth or e-health or "e health" or telecare or tele-care or "tele care" or telemonitor* or tele-monitor* or "tele monitor*" or teleconsult* or tele-consult* or "tele consult*" or "remote health*" or "remote care" or "remote medic*" or "remote monitor*" or "remote consult*" or "virtual health*" or "virtual care" or "virtual medic*" or "virtual monitor*" or "virtual consult*" or "distant health*" or "distant care" or "distant medic*" or "distant monitor*" or "distant consult*" or "distance health*" or "distance care" or "distance medic*" or "distance monitor*" or "distance consult*").ab,ti. | 78,583 |
| 2 | telemedicine/ or teleconsultation/ or telemonitoring/ or video consultation/ | 46,629 |
| 1 | (elder* or senior? or geriatric? or "older person?" or "older people" or "old person?" or "old people" or gerontolog* or ageing or aging).ab,ti. | 782,753 |

Search strategy and result for PubMed (searched on 2021 Sep 16th)

| **Search number** | **Query** | **Filters** | **Results** |
| --- | --- | --- | --- |
| 6 | #1 AND #4 | English | 3,192 |
| 5 | #1 AND #4 |  | 3,315 |
| 4 | #2 OR #3 |  | 77,483 |
| 3 | telehealth[Title/Abstract] or tele-health[Title/Abstract] or "tele health"[Title/Abstract] or telemedicine[Title/Abstract] or tele-medicine[Title/Abstract] or "tele medicine"[Title/Abstract] or "mobile health"[Title/Abstract] or mHealth[Title/Abstract] or "m health"[Title/Abstract] or m-health[Title/Abstract] or "electronic health"[Title/Abstract] or eHealth[Title/Abstract] or e-health[Title/Abstract] or "e health"[Title/Abstract] or telecare[Title/Abstract] or tele-care[Title/Abstract] or "tele care"[Title/Abstract] or telemonitor*[Title/Abstract] or tele-monitor*[Title/Abstract] or "tele monitor*"[Title/Abstract] or teleconsult*[Title/Abstract] or tele-consult*[Title/Abstract] or "tele consult*"[Title/Abstract] or "remote health*"[Title/Abstract] or "remote care"[Title/Abstract] or "remote medic*"[Title/Abstract] or "remote monitor*"[Title/Abstract] or "remote consult*"[Title/Abstract] or "virtual health*"[Title/Abstract] or "virtual care"[Title/Abstract] or "virtual medic*"[Title/Abstract] or "virtual monitor*"[Title/Abstract] or "virtual consult*"[Title/Abstract] or "distant health*"[Title/Abstract] or "distant care"[Title/Abstract] or "distant medic*"[Title/Abstract] or "distant monitor*"[Title/Abstract] or "distant consult*"[Title/Abstract] or "distance health*"[Title/Abstract] or "distance care"[Title/Abstract] or "distance medic*"[Title/Abstract] or "distance monitor*"[Title/Abstract] or "distance consult*"[Title/Abstract] |  | 66,910 |
| 2 | Telemedicine[MeSH:NoExp] |  | 30,050 |
| 1 | elder*[Title/Abstract] OR senior[Title/Abstract] OR seniors[Title/Abstract] OR geriatric[Title/Abstract] OR geriatrics[Title/Abstract] OR "older person"[Title/Abstract] OR "older persons"[Title/Abstract] OR "older people"[Title/Abstract] OR "old person"[Title/Abstract] OR "old persons"[Title/Abstract] OR "old people"[Title/Abstract] OR gerontolog*[Title/Abstract] OR ageing[Title/Abstract] OR aging[Title/Abstract] |  | 618,660 |

Search strategy and result for Cochrane Library (searched on 2021 Sep 16th)

| **ID** | **Search** | **Hits** |
| --- | --- | --- |
| 5 | #1 AND #4 | 148 |
| 4 | #2 OR #3 | 13,143 |
| 3 | (telehealth or tele-health or "tele health" or telemedicine or tele-medicine or "tele medicine" or "mobile health" or mHealth or "m health" or m-health or "electronic health" or eHealth or e-health or "e health" or telecare or tele-care or "tele care" or telemonitor* or tele-monitor* or "tele monitor*" or teleconsult* or tele-consult* or "tele consult*" or "remote health*" or "remote care" or "remote medic*" or "remote monitor*" or "remote consult*" or "virtual health*" or "virtual care" or "virtual medic*" or "virtual monitor*" or "virtual consult*" or "distant health*" or "distant care" or "distant medic*" or "distant monitor*" or "distant consult*" or "distance health*" or "distance care" or "distance medic*" or "distance monitor*" or "distance consult*"):ti,ab,kw (Word variations have been searched) | 13,143 |
| 2 | MeSH descriptor: [Telemedicine] this term only | 2,444 |
| 1 | ("artificial intelligence" or "Computational Intelligence" or "Computer Reasoning" or "Computer Vision System*" or "Knowledge Acquisition" or "Knowledge Representation" or "Machine Intelligence" or "machine learning" or "transfer learning" or "deep learning" or "Hierarchical Learning" or "computer-assisted diagnosis" or "computer assisted diagnosis"):ti,ab,kw (Word variations have been searched) | 2,922 |

Search strategy and result for CINAHL (searched on 2021 Sep 16th)

| **#** | **Query** | **Limiters/Expanders** | **Last Run Via** | **Results** |
| --- | --- | --- | --- | --- |
| S6 | S1 AND S4 | Expanders - Apply equivalent subjects  Narrow by Language: - english  Search modes - Find all my search terms | Interface - EBSCOhost Research Databases  Search Screen - Advanced Search  Database - CINAHL Complete | 1,488 |
| S5 | S1 AND S4 | Expanders - Apply equivalent subjects  Search modes - Find all my search terms | Interface - EBSCOhost Research Databases  Search Screen - Advanced Search  Database - CINAHL Complete | 1,523 |
| S4 | S2 OR S3 | Expanders - Apply equivalent subjects  Search modes - Find all my search terms | Interface - EBSCOhost Research Databases  Search Screen - Advanced Search  Database - CINAHL Complete | 43,631 |
| S3 | TI ( telehealth or tele-health or "telehealth" or telemedicine or tele-medicine or "tele medicine" or "mobile health" or mHealth or "m health" or m-health or "electronic health" or eHealth or e-health or "e health" or telecare or tele-care or "tele care" or telemonitor* or tele-monitor* or "tele monitor*" or teleconsult* or tele-consult* or "tele consult*" or "remote health*" or "remote care" or "remote medic*" or “remote monitor*" or "remote consult*" or "virtual health*" or “virtual care" or "virtual medic*" or “virtual monitor*" or "virtual consult*" or "distant health*" or “distant care" or "distant medic*" or “distant monitor*" or "distant consult*" or "distance health*" or “distance care" or "distance medic*"or "distance monitor*" or "distance consult*" ) OR AB ( telehealth or tele-health or "tele health" or telemedicine or tele-medicine or “tele medicine" or "mobile health" or mHealth or "m health" or m-health or "electronic health" or eHealth ore-health or "e health" or telecare or tele-care or "tele care" or telemonitor* or tele-monitor* or "telemonitor*" or teleconsult* or tele-consult* or "tele consult*" or “remote health*" or "remote care" or “remote medic*" or "remote monitor*" or "remote consult*" or “virtual health*" or "virtual care" or “virtual medic*" or "virtual monitor*"or "virtual consult*" or "distant health*" or "distant care" or "distant medic*" or "distant monitor*" or “distant consult*" or "distance health*" or "distance care" or “distance medic*" or "distance monitor*" or "distance consult*" ) | Expanders - Apply equivalent subjects  Search modes - Find all my search terms | Interface - EBSCOhost Research Databases  Search Screen - Advanced Search  Database - CINAHL Complete | 35,980 |
| S2 | (MH "Telemedicine") OR (MH "Remote Consultation") | Expanders - Apply equivalent subjects  Search modes - Find all my search terms | Interface - EBSCOhost Research Databases  Search Screen - Advanced Search  Database - CINAHL Complete | 15,745 |
| S1 | TI ( elder* or senior# or geriatric# or “older person#" or "older people" or “old person#" or "old people" or gerontolog* or ageing or aging ) OR AB ( elder* or senior# or geriatric# or "older person#" or "older people" or "old person#" or "old people" or gerontolog* or ageing or aging ) | Expanders - Apply equivalent subjects  Search modes - Find all my search terms | Interface - EBSCOhost Research Databases  Search Screen - Advanced Search  Database - CINAHL Complete | 229,269 |

Search strategy and result for PsycInfo (EBSCO) (searched on 2021 Sep 16th)

| **#** | **Query** | **Limiters/Expanders** | **Last Run Via** | **Results** |
| --- | --- | --- | --- | --- |
| S6 | S1 AND S4 | Expanders - Apply equivalent subjects  Narrow by Language: - english  Search modes - Find all my search terms | Interface - EBSCOhost Research Databases  Search Screen - Advanced Search  Database - APA PsycInfo | 705 |
| S5 | S1 AND S4 | Expanders - Apply equivalent subjects  Search modes - Find all my search terms | Interface - EBSCOhost Research Databases  Search Screen - Advanced Search  Database - APA PsycInfo | 733 |
| S4 | S2 OR S3 | Expanders - Apply equivalent subjects  Search modes - Find all my search terms | Interface - EBSCOhost Research Databases  Search Screen - Advanced Search  Database - APA PsycInfo | 15,083 |
| S3 | TI ( telehealth or tele-health or "telehealth" or telemedicine or tele-medicine or "tele medicine" or "mobile health" or mHealth or "m health" or m-health or "electronic health" or eHealth or e-health or "e health" or telecare or tele-care or “tele care" or telemonitor* or tele-monitor* or "tele monitor*" or teleconsult* or tele-consult* or "tele consult*" or "remote health*" or “remote care" or "remote medic*" or “remote monitor*" or "remote consult*" or "virtual health*" or “virtual care" or "virtual medic*" or “virtual monitor*" or "virtual consult*" or "distant health*" or “distant care" or "distant medic*" or “distant monitor*" or "distant consult*" or "distance health*" or “distance care" or "distance medic*"or "distance monitor*" or "distance consult*" ) OR AB ( telehealth or tele-health or "tele health" or telemedicine or tele-medicine or “tele medicine" or "mobile health" or mHealth or "m health" or m-health or "electronic health" or eHealth ore-health or "e health" or telecare or tele-care or "tele care" or telemonitor* or tele-monitor* or "telemonitor*" or teleconsult* or tele-consult* or "tele consult*" or “remote health*" or "remote care" or “remote medic*" or "remote monitor*" or "remote consult*" or “virtual health*" or "virtual care" or “virtual medic*" or "virtual monitor*"or "virtual consult*" or "distant health*" or "distant care" or "distant medic*" or "distant monitor*" or “distant consult*" or "distance health*" or "distance care" or “distance medic*" or "distance monitor*" or "distance consult*" ) | Expanders - Apply equivalent subjects  Search modes - Find all my search terms | Interface - EBSCOhost Research Databases  Search Screen - Advanced Search  Database - APA PsycInfo | 8,956 |
| S2 | DE "Telemedicine" OR DE "Online Therapy" OR DE "Teleconsultation" | Expanders - Apply equivalent subjects  Search modes - Find all my search terms | Interface - EBSCOhost Research Databases  Search Screen - Advanced Search  Database - APA PsycInfo | 10,219 |
| S1 | TI ( elder* or senior# or geriatric# or “older person#" or "older people" or “old person#" or "old people" or gerontolog* or ageing or aging ) OR AB ( elder* or senior# or geriatric# or "older person#" or "older people" or "old person#" or "old people" or gerontolog* or ageing or aging ) | Expanders - Apply equivalent subjects  Search modes - Find all my search terms | Interface - EBSCOhost Research Databases  Search Screen - Advanced Search  Database - APA PsycInfo | 167,288 |
