# Supplementary material for: Domains, Feasibility, Effectiveness, Cost, and Acceptability of Telehealth in Aging Care: Scoping Review of Systematic Reviews
Source: JMIR Aging. 2023 Apr 18;6:e40460. doi: 10.2196/40460 (PMC10155091; doi:10.2196/40460)
Supplement: Multimedia Appendix 2 [file aging_v6i1e40460_app2.docx]

Multimedia Appendix 2. Characteristics of the included reviews

| REFERENCES | TYPE AND AIM OF THE SYSTEMATIC REVIEW | NO. OF DATABASES SEARCHED & ARTICLES INCLUDED | LOCATIONS OF THE STUDIES CONDUCTED REPORTED | LANGUAGE AND DATE RANGE OF SEARCH | INCLUDED STUDY DESIGNS |
| --- | --- | --- | --- | --- | --- |
| Inglis et al.  (2015)  [29] | Post-hoc sub-analysis of a previously published large Cochrane systematic review and meta-analysis  To determine whether structured telephone support and telemonitoring were effective in older people with heart failure | 1 Cochrane systematic review and meta-analysis of 25 articles |  |  | RCTs^a^ |
| Jones et al.  (2002)  [62] | Integrated literature review  To examine Telehealth technologies used in the clinical nursing care of elders, and to determine their value in supporting nursing functions (not only to determine how these technological innovations support the nursing care of elders but also to examine the extent to which these technologies offer support for the professional dimensions of gerontological practice) | 5 databases  18 articles included |  |  | Exploratory or experimental study, specifically exploring the association between one intervention variable and the technology used is incorporated in the research design. |
| Barlow et al.  (2007)  [63] | Systematic review  *(The heterogeneity in design, participants and intervention types made a meta-analysis quantifying the effects of different interventions impossible.)*  To examine the potential effects of telecare in terms of   (a) benefits to individuals,  (b) system-wide benefits, such as effects on costs, and care processes. | 17 databases  98 articles included | US (64%) UK (10%) |  | RCTs^1^ of any size and observational studies with 80 or more participants |
| Franek  (2012)  [64] | Systematic review  To conduct an evidence-based assessment of home telehealth technologies for patients with COPD^b^ in order to inform recommendations regarding the access and provision of these services in Ontario | 6 databases  RQ1: 6 articles included  RQ2: 1 article included |  | English  January 1, 2000 - November 3, 2010 | RCTs^a^, CCTs^c^, systematic reviews, and/or meta-analyzes; |
| van den Berg et al.  (2012)  [70] | Systematic review  To answer the following research questions:  (1) Is it feasible to support healthcare of older patients with chronic diseases in their homes with telemedical systems? (2) For which specific diseases/disease groups have telemedicine healthcare concepts been developed? (3) What are the respective applications, modalities, and target parameters of telemedicine interventions? (4) Can determinants of successful telemedicine applications be identified? (5) How good is the acceptance of telemedicine among older patients and among their caregivers? Can specific factors be identified which positively or adversely influence acceptance? | 3 databases  68 articles included | US (32) Europe (26) Canada (6) Israel (2) Australia (1) Argentine (1) | English  2007 - February 3, 2012  This restriction of the time period reduces heterogeneity due to technical development and communication infrastructure thus improving comparability between the studies. | Controlled design and analyzes for a minimum number of five participants in the targeted age range of 60 years and older |
| Foster et al.  (2014)  [71] | Integrative review  To  (1) describe current telehealth devices used to transmit physiological data in older adults with chronic illness   (2) evaluate facilitators of and barriers to telehealth technologies available to elderly patients with chronic physical disease. | 3 databases  14 articles included | US (7) Netherlands (2) UK (1) Germany (1) Australia (1) France (1) Denmark (1) Norway (1) Canada (1) | English  between January 2003 and December 2013 |  |
| Nordheim et al.  (2014)  [72] | Systematic review  To assess whether telemedicine follow-up care of patients with leg and foot ulcers, specifically the transfer of digital still images or video consultations, affects clinical, behavioral or organizational outcomes compared with traditional follow-up care. | 5 databases  1 article included | US (1) | English, Norwegian, Swedish and Danish  After 1980 | RCTs^a^, non-randomized trials, controlled before-after studies and prospective cohort studies with a comparison of treated and non-treated groups. |
| Peretz et al.  (2016)  [73] | Systematic review  To review the cost of RPM^i^ programs targeting elderly patients with chronic conditions. | 6 databases  13 articles included | USA (5) UK (2) New Zealand (1) Netherlands (1) Italy (1) Canada (1) | English | Primary studies |
| Frost et al.  (2017)  [74] | Systematic review with meta-analysis  To synthesize evidence from RCTs^1^ to evaluate the effectiveness of home- and community-based health promotion interventions on functioning and frailty in older people with mild or pre-frailty. | 23 databases  10 articles included | US (4) Germany (1) Japan (1) Brazil (1) | English  January 1990 - May 2016 | Randomized controlled parallel-group or crossover trials |
| Karlsen et al.  (2017)  [75] | Qualitative systematic review  To identify and synthesize the best available qualitative evidence of community-dwelling older adults’ experience with the use of telecare in home care services. | 4 databases  11 articles included | Spain (3) UK (3) US (2) Netherlands (1) Sweden (1) | English, Norwegian, Swedish, Danish  2005 - 2017 | Primary studies that focused on qualitative data including, but not limited to, designs such as phenomenology, grounded theory, ethnography, action research and feminist research. Mixed method studies with a qualitative component were also included when appropriate. |
| Narasimha et al.  (2017)  [76] | Systematic review  To understand  (1) the characteristics of usability-related studies conducted with the geriatric population,  (2) the aspects of usability explored in these articles and the instruments used to measure them,  (3) the data analysis methods used,  (4) the impact of the usability of geriatric telemedicine technology on the geriatric user population,  (5) usability challenges associated with telemedicine platforms,  (6) the limitations observed. | 2 databases  16 articles included | US (7) China (3) Canada (1) Netherlands (1) Germany (1) France (1) Denmark (1) Singapore (1) | English  2000 - July 2016 | Pilot study |
| Gentry et al.  (2018)  [77] | Systematic review  To discuss the available literature on feasibility, acceptability, and cost-effectiveness of psychiatric assessment and treatment modalities within TMH^j^ for geriatric patients. | 4 databases  76 articles included | US (35) Canada (10) Australia (10) China (including HK) (4) France (3) Italy (3) UK (2) South Korea (1) | English  1990 - August 6th, 2018 |  |
| Marx et al.  (2018)  [65] | Systematic review with meta-analysis  To determine the efficacy of telehealth methods in delivering malnutrition-related interventions to community-dwelling older adults. | 5 databases  13 articles included |  | Any language | Original research |
| Santana et al.  (2018)  [66] | Systematic review  To analyze the available literature on the use of telecare as a nursing intervention in care for the elderly with Alzheimer's and their caregivers. | 4 databases  2 articles included |  | Portuguese, English and Spanish languages  2007 - 2017 | Cohort studies and randomized controlled trials with allocation confidentiality |
| Batsis et al.  (2019)  [67] | Systematic review  *(Significant methodological heterogeneity precluded meta-analysis)*  To conduct a systematic evaluation of the evidence regarding TMed^d^ interventions conducted in older adults in non-hospital settings. | 6 databases  17 articles included |  | English  January 2012 - July 2018 | Only RCTs^a^ (human studies) |
| Christensen et al.  (2019)  [68] | Systematic review  *(Because the quality of the articles was not good enough - as judged by the CASP^e^ criteria - we did not conduct a meta-analysis.)*  To conduct a systematic review of the existing research literature, focusing on patients' and providers' experiences of VCs^f^ used in the treatment of patients 60+ years with unipolar depression. | 8 databases  21 articles included | US (12) Canada (6) Spain (1) Australia (1) HK (1) Germany (1) | English, German, Danish, Norwegian and Swedish  January 2000 - December 2017 | Studies using qualitative, quantitative or combined quantitative/qualitative methods, stating an aim, method, results and a conclusion were all included |
| Costanzo et al.  (2019)  [69] | Systematic literature review  To provide an updated narrative synthesis of the scientific literature about the implementation of telemedicine for diagnosis, treatment, and follow‐up of patients with AD^g^ and MCI^h^ and their caregivers. | 3 databases  56 articles included | US (17) Italy (14) China (4) France (3) Australia (3) Spain (2) Greece (1) Brazil (1) Sweden (1) Germany (1) Netherlands (1) | English | All study designs |
| Aquilanti et al.  (2020)  [78] | Systematic review  *(A meta-analysis was not performed in order to avoid the systematic error that could have occurred due to the selection and publication bias and the heterogeneity among the studies.)*  To assess the feasibility (accuracy and the effectiveness of Teledentistry compared to traditional face-to-face dental visits, the patient acceptability, and the costs related to the implementation of oral health information technology provision) of Teledentistry in communities or in a domiciliary setting where elderly people live | 5 databases  6 articles included | Australia (4) France (2) Germany (1) | English | Clinical studies |
| Kruse et al.  (2020)  [79] | Systematic review  To evaluate the current literature to help identify and understand health-related quality of life enhancers and general health outcomes that are commensurate with and barriers to the use of telehealth services by older adults. | 4 databases  57 articles included |  | English  in the last 5 years |  |
| Sekhon et al.  (2021)  [82] | Systematic review  To examine the impact of telemedicine on health outcomes in elderly individuals with dementia living in rural areas. | 3 databases  12 articles included |  |  | Original studies |
| Elbaz et al.  (2021)  [83] | Systematic review  To explore the use, feasibility, and acceptability of telemedicine applications for older adults with dementia during the COVID-19 pandemic to address these gaps, as well as examine the potential mental health impacts of these technologies | 3 databases  7 articles included | US (2) Italy (1) China (1) Spain (1) France (1) UK (1) | English  2020 - October 2021 |  |
| Tam et al.  (2022)  [84] | Systematic review  To identify the effects of TMIs^k^ on hypertension management among OAwHTN^l^ in terms of frequency, directionality, and content. | 6 databases  6 articles included | China (4) Chile (1) Sweden (1) | Chinese and English  January 1, 2010 - December 31, 2020 | RCTs^a^ were chosen to provide reliable evidence regarding the effects of TMI^k^. |
| Markert et al.  (2021)  [85] | Systematic literature review  To investigate the current status of health coaching interventions incorporating telehealth technology and the associated effectiveness of this intervention to deliver health care with an emphasis on older adults (aged 65 and older). | 2 databases  13 articles included |  | English  January 2010-September 5, 2019 |  |
| Wong et al.  (2022)  [86] | Systematic review with meta-analysis  To summarize findings from randomized controlled trials on the effect of nurse-led telehealth self-care promotion programs compared with the usual on-site or face-to-face services on QoL^m^, self-efficacy, depression, and hospital admissions among community-dwelling older adults. | 6 databases  13 articles included | US (5) HK (3) Australia (2) UK (1) Japan (1) Finland (1) | English  May 2011 - April 2021 |  |
| Rush et al.  (2022)  [87] | Systematic Mixed Studies Review  To synthesize evidence for use of telehealth to promote health among rural-living older adults, as well as to explore cost-effectiveness for the health care system and older adults. | 5 databases  42 articles included |  | English |  |
| Haimi et al.  (2022)  [88] | Systematic review  To explore the availability, application, and implementation of telehealth services during the Covid-19 era, designed for the aged population (age 65 and more). | 3 databases  11 articles included | Singapore (2) US (2) Italy (2) Poland (1) Tunisia (1) Turkey (1) Netherlands (1) Ireland (1) | English  December 2019 - 27 December 2020 |  |
| Pool et al.  (2022)  [89] | Integrative review  To address the research questions: "how do data privacy issues influence the adoption and use of telehealth in aged care contexts" | 4 databases  16 articles included | US (5) UK (4) Brazil (1) Ireland (1) Netherlands (1) Finland (1) Sweden (1) Greece (1) Taiwan (1) | English |  |
| Al-Naher et al.  (2022)  [80] | Systematic review  To explore the use of remote distance technologies in heart failure. | 5 databases  52 articles included | US (18) UK (12) Canada (8) Sweden (7) Mexico (1) Denmark (1) Switzerland (1) Scotland (1) Germany (1) New Zealand (1) Wales (1) Australia (1) | English  January 1, 1990 - September 19, 2020 |  |
| Murphy et al.  (2020)  [81] | Rapid review  *(There was considerable heterogeneity in data reporting with variability of methodology in the studies, so a formal meta-analysis could not be conducted.)*  To describe the acceptability of a telehealth service as a model of care for geriatric outpatient consultations, with secondary aims of evaluating the productivity, clinical benefit, costs and challenges encountered with such services. | 3 databases  9 articles included | US (5) Canada (2) HK (1) Australia (1) |  | Studies of any design |

*^a^RCT: Randomized Controlled Trial, ^b^COPD: Chronic Obstructive Pulmonary Disease, ^c^CCT: Controlled Clinical Trial, ^d^TMed: Telemedicine, ^e^CASP: Critical Appraisal Skills Program, ^f^VC: Videoconferencing, ^g^AD: Alzheimer's Disease, ^h^MCI: Mild Cognitive Impairment, ^i^RPM: Remote Patient Monitoring, ^j^TMH: Telemental Health, ^k^TMI: Text Messaging Intervention, ^l^OAwHTN: Older Adults with Hypertension, ^m^QoL: Quality of Life*
