# Supplementary material for: Domains, Feasibility, Effectiveness, Cost, and Acceptability of Telehealth in Aging Care: Scoping Review of Systematic Reviews
Source: JMIR Aging. 2023 Apr 18;6:e40460. doi: 10.2196/40460 (PMC10155091; doi:10.2196/40460)
Supplement: Multimedia Appendix 3 [file aging_v6i1e40460_app3.docx]

Multimedia Appendix 3. Summary of the conceptual and operational definitions of the terminologies

| **TERMINOLOGY** | **REFERENCE** | **CONCEPTUAL DEFINITION** | **OPERATIONAL DEFINITION** |
| --- | --- | --- | --- |
| TELEHEALTH | Jones et al.  (2002)  [62] | The use of telecommunications and interactive technologies to provide health information and services is known as Telehealth.  Telehealth is defined as the use of interactive technology for the provision of clinical health care, patient and professional education, and health care administration over small and large distances [30]. The defining aspect of Telehealth is the use of electronic signals to transfer various types of information from one site to another. Information ranges from clinical records to health promotion instructions to still-images of wound and motion-images demonstrating exercise routines.  The term Telehealth is embraced as the more encompassing concept, descriptive of the state of technology use in the provision of health care; telemedicine and telenursing are subsets of Telehealth. | Telehealth is broadly defined as the use of interactive computer or communications technology for the provision of health care over a small or long distance.  This review of Telehealth applications will focus only on the application of Telehealth to facilitate the professional-patient relationship. |
|  | Franek  (2012)  [64] |  | Telemedicine (or telehealth) refers to using advanced information and communication technologies and electronic medical devices to support the delivery of clinical care, professional education, and health-related administrative services.  Telehealth is often associated with a broader definition of remote health care and is perceived to be more focused on other health-related services. |
|  | Foster et al.  (2014)  [71] | Telehealth is a term used to describe the use of telecommunication devices that allow for the synchronous or asynchronous exchange of healthcare information between geographically separated individuals [32].  Telehealth, by definition, is the use of various forms of communication technologies (television, e-mail, telephone, videoconferencing, Internet, radio) and the electronic exchange of healthcare information to provide long-distance clinical healthcare to patients. |  |
|  | Marx et al.  (2018)  [65] | Telehealth can be defined as the delivery of healthcare services from a distance using telecommunication techniques synchronously (i.e. same time, different location) and/or asynchronously (i.e. different time, different location) [31]. | Telehealth was considered as: 1) a synchronistic consultation with a health professional with point-of-contact via any telephone or internet-based method, or 2) an asynchronistic telephone- or internet-based intervention system. |
|  | Markert et al.  (2021)  [85] | Telehealth is an all-encompassing term for clinical and nonclinical remote health care services and is defined by the Centre for Connected Health Policy as “a collection of means or methods for enhancing health care, public health and health education delivery and support using telecommunications technologies” [33]. | For the purpose of this literature review, telehealth includes telemedicine, remote patient monitoring (RPM), remote activity monitoring (RAM), decision support systems (DSSs), and health coaching systems. |
|  | Wong et al.  (2022)  [86] | Telehealth refers to the services that bring health care directly to users, generally in their own homes, supported by information and communication technology [34].  It includes but is not limited to social alarms, lifestyle monitoring, remote monitoring of vital signs for diagnosis, and long-distance assessment and education. | Defined as the use of apps, websites, WhatsApp, SMS text messages, email, social media such as Facebook or Twitter, telephone calls, tablets, software such as Zoom or Microsoft Teams, home remote monitoring devices [reactive or proactive], or any combination of these as a health care delivery channel |
|  | Rush et al.  (2022)  [87] | Telehealth refers to the provision of health services, education, and support from a distance using a variety of technologies including telephone, email, video (synchronous/asynchronous), and smartphone applications [35]. |  |
|  | Haimi et al.  (2022)  [88] | Telehealth is the delivery of healthcare services by healthcare professionals through information and communication technologies (ICT), where distance separates between the participants [36].  In the context of the COVID-19 pandemic, telehealth has been used broadly as an aid to the active management of patients with COVID-19, for surveillance, triage, and diagnosis; treatment including e-prescriptions; follow-up care; and rehabilitation.  Telehealth has been also complemented by the use of wearable devices and self-care equipment, such as glucometers, handheld blood pressure monitors, pulse oximeters, and digital stethoscopes [37]. | As cited by Shaw, “Telehealth is the use of electronic communications to provide and deliver a host of health-related information and health care services, including, but not limited to physical therapy-related information and services, over large and small distances. Telehealth encompasses a variety of health care and health promotion activities, including, but not limited to, education, advice, reminders, interventions, and monitoring of interventions.” [36]  Telehealth applications include live (synchronous) videoconferencing [a two-way audiovisual link between a patient and a care provider]; store-and-forward (asynchronous) videoconferencing [transmission of a recorded health history to a health practitioner, usually a specialist]; remote patient monitoring (RPM) [the use of connected electronic tools to record personal health and medical data in one location for review by a provider in another location, usually at a different time]; and mobile health (mHealth) [health care and public health information provided through mobile devices]. |
| TELEMEDICINE | Franek  (2012)  [64] |  | Telemedicine (or telehealth) refers to using advanced information and communication technologies and electronic medical devices to support the delivery of clinical care, professional education, and health-related administrative services. Telemedicine is often associated with direct patient clinical services |
|  | van den Berg et al.  (2012)  [70] | The American Telemedicine Association (ATA) defines telemedicine as “the use of medical information exchanged from one site to another via electronic communications to improve patients’ health status”. In this definition, telemedicine is confined to its core function as immediate medical service. Other terms such as “telehealth” or “e-health” are broader and include also related services, e.g., patient portals or educational and training services. |  |
|  | Nordheim et al.  (2014)  [72] | Telemedicine is defined as “the use of electronic information and communication technologies to provide and support health care when distance separates the participants” [41]. |  |
|  | Karlsen et al.  (2017)  [75] |  | Telemedicine can be seen as the remote diagnosis, monitoring and management of patients’ medical conditions [42]. |
|  | Narasimha et al.  (2017)  [76] | Telemedicine, which is the exchange of medical information between locations through the use of electronic communication devices, is primarily used to improve healthcare services [43].  Its most significant feature, its dependence on communication technology, allows it to function as a surrogate to in-person meetings [43], thus providing diagnosis, pretreatment and/or posttreatment to medical issues comparable to the conventional face-to-face method of medical practice [44]. |  |
|  | Gentry et al.  (2018)  [77] | Telemedicine, defined as providing healthcare at a distance through the use of telecommunications technology [45]. |  |
|  | Batsis et al.  (2019)  [67] | Telemedicine or telehealth encompasses many different modalities of using technology to deliver care, synchronous, two-way video-conferencing (referred and defined in this manuscript as telemedicine or TMed) [38-40]. | TMed was defined as live, real-time, synchronous, two-way video-conferencing on both the receiving and delivery end, as this is the most common type used within clinical settings and one that is most fully reimbursed |
|  | Kruse et al.  (2020)  [79] | The WHO defines telemedicine as “healing from a distance.” More specifically, it is healing through the use of information and communication technologies “to improve patient outcomes by increasing access to care and medical information” [31].  The WHO also does not differentiate between the terms telemedicine and telehealth. |  |
|  | Elbaz et al.  (2021)  [83] | Telemedicine, an approach that incorporates information and communication technologies in the delivery of health care services for the diagnosis, treatment, prevention, and research and evaluation in order to advance patients’ health outcomes [46]. |  |
|  | Markert et al.  (2021)  [85] | Telemedicine is the use of telecommunication technology to allow health care workers to provide clinical services (e.g., medical therapy) to patients remotely [47]. |  |
|  | Haimi et al.  (2022)  [88] | Telemedicine is a term referring specifically to the remote clinical services [48]. | Telemedicine technology is a subset of health IT that refers specifically to remote clinical services. |
|  | Murphy et al.  (2020)  [81] | Telemedicine is the exchange of medical information between locations through the use of electronic communication devices and it aims to function as a surrogate to in-person consultations [43]. |  |
| TELECARE | Barlow et al.  (2007)  [63] | Terms such as telecare, telemedicine and telecare are often used interchangeably. | Telecare is defined as the use of communications technology to provide health and social care directly to the user (patient).   This excludes the exchange of information solely between professionals, generally for diagnosis or referral. There are differences between these functions in terms of their structural and operational complexity [49], with implications for widespread implementation. |
|  | van den Berg et al.  (2012)  [70] | Telemedical connections between healthcare providers and individual patients in their homes, often referred to as “telecare” [50]. | We define telecare as “remote treatment”, which includes concepts based on dedicated technical devices as well as concepts which use conventional techniques such as telephone contacts or short messages. |
|  | Karlsen et al.  (2017)  [75] | Technology referred to as ‘‘telecare’’ is defined as the use of communication technology to provide health and social care directly to the user [63]. | **Telecare** is an umbrella concept comprising several technological solutions that promote safety and security in people’s homes [63].  It can be classified into three categories:   - first generation devices are the simplest form of telecare and include a user triggered alarm button (active alarms); - second-generation telecare systems utilize a range of sensors that detect specific hazards, and they do not require the users to trigger them (passive alarms); - third generation telecare systems have more complex capabilities that constitute ‘‘lifestyle monitoring’, where data are sent to an internet portal that can be assessed by caregivers who are ‘‘keeping an eye’’ on the user [51]. |
|  | Santana et al. (2018)  [66] | Telecare is an integrated system of health activities that can be carried out at a distance. |  |
| STRUCTURED TELEPHONE | Inglis et al. (2015)  [29] |  | Remote heart failure monitoring (via structured telephone support or telemonitoring) occurring on a regular schedule (daily, weekly, or monthly). The remote monitoring had to be initiated by a healthcare professional (for example, medical, nursing, social work, pharmacist) and must have been delivered as the only heart failure disease management intervention, without home-visits or intensified clinic follow-up. |
| TELEPSYCHIATRY | Christensen et al.  (2019)  [68] | Telepsychiatry has been explored as an alternative to the traditional modes and can be broadly defined as mental health care at a distance [52,53]. | Telepsychiatry is defined as the provision of mental health services via video consultation technology [54]. |
| TELEDENTISTRY | Aquilanti et al.  (2020)  [78] | Teledentistry is defined as the use of health information technology and telecommunications for oral care [55,56]. |  |
| TELEMONITORING | Franek  (2012)  [64] |  | Telemonitoring (or remote monitoring) refers to the use of medical devices to remotely collect a patient’s vital signs and/or other biologic health data and the transmission of those data to a monitoring station for interpretation by a health care provider. |
| TELEPHONE ONLY SUPPORT | Franek  (2012)  [64] |  | Telephone only support refers to disease/disorder management support provided by a health care provider to a patient who is at home via telephone or videoconferencing technology in the absence of transmission of patient biologic data. |
| TELENURSING | Franek  (2012)  [64] |  | Telenursing generally refers to the in-person visit of a health care provider, typically a nurse, to a patient’s home or residence, regularly, in order to provide clinical care or professional education. |
| HEALTH INFORMATION TECHNOLOGY | Haimi et al.  (2022)  [88] | The term “health information technology” (health IT) is a broad category of solutions that includes technologies to store, share, and analyze health information. |  |
| REMOTE CARE PROGRAMS | Al-Naher et al.  (2022)  [80] |  | Remote care programs are defined as any intervention accessible from the patient’s home or local community, which provides the patient with education, assessment, investigation results, or otherwise replaces a service that would normally be offered within a formal clinical setting. |
| REMOTE PATIENT MONITORING (RPM) | Peretz et al.  (2016)  [73] | Remote patient monitoring (RPM), consists of the transmission of healthcare data from a patient to a healthcare provider in different locations for assessment and intervention purposes and is used primarily to support the care and self-management of patients with chronic conditions. |  |
|  | Markert et al.  (2021)  [85] | RPM is the use of electronic devices and telecommunication technology to monitor and transmit patient physiological or metabolic parameters to a digital database that can be accessed by authorized users [57].  RPM usually involves Bluetooth-enabled or internet-connected devices that automatically transmit monitored parameters. RPM can also include electronic wellness questionnaires that elicit information concerning the patient’s well-being and health status. |  |
| REMOTE ACTIVITY MONITORING (RAM) | Markert et al.  (2021)  [85] | RAM is the use of electronic devices to provide remote monitoring of a person’s mobility or activities of daily living (ADLs) [58].  ADLs can be remotely monitored using motion detection devices installed in a person’s residence or a wearable device, such as a smart watch, that detects, records, and transmits movement activity. Another form of ADL monitoring is medication adherence monitored remotely via automated pillboxes. Automated pillboxes are used to organize medications, provide reminders to take medications, and provide information to clinicians via telehealth regarding medication use [59]. |  |
| DECISION SUPPORT SYSTEMS (DSSs) | Markert et al.  (2021)  [85] | DSSs are electronic (computerized) systems which evaluate data collected via remote monitoring and transform the data into useful information regarding the patient’s health and wellness [60].  The DSS makes clinical or behavioral recommendations based on an evaluation of the monitored data. An example of a recommendation is a reminder to the patient to take his/her medication if an automated pillbox senses the person has not taken their medication that day. If the medication is still not taken after some delay, the DSS can notify the health care providers or health coaching system. The DSS can also initiate an emergency notification to 911 if certain threshold values of monitored parameters are exceeded. |  |
| HEALTH COACHING SYSTEMS | Markert et al.  (2021)  [85] | Health coaching systems are defined as “patient-centered processes that are based upon behavior change theory” and include goal setting, education, encouragement, and feedback on health-related behaviors [61].  Health coaching programs provide health-related information, recommendations, or encouragement to the patient on a routine or as-needed basis to help drive behavior changes [60].  Forms of health coaching include encouragement, feedback, health care suggestions, periodic health tips, or short educational presentations based on an analysis of the patient’s health status and monitored data.  The health coaching system can be manual (human health coach only), partially automated, or fully automated using artificial intelligence and machine learning to generate health coaching messages to the patient. |  |
