# Supplementary material for: Domains, Feasibility, Effectiveness, Cost, and Acceptability of Telehealth in Aging Care: Scoping Review of Systematic Reviews
Source: JMIR Aging. 2023 Apr 18;6:e40460. doi: 10.2196/40460 (PMC10155091; doi:10.2196/40460)
Supplement: Multimedia Appendix 4 [file aging_v6i1e40460_app4.docx]

Multimedia Appendix 4. Inclusion and exclusion criteria of the included reviews

| References | Population | Intervention & Comparator | Exclusion criteria |
| --- | --- | --- | --- |
| Inglis et al.  (2015)  [29] | - Adult patients with a confirmed diagnosis of heart failure who were discharged from an acute care setting to home (excluding nursing or convalescent homes) - Adult patients who were managed for heart failure in the community setting | **Intervention:** Remote heart failure monitoring (via structured telephone support or telemonitoring)   - occurring on a regular schedule (daily, weekly, or monthly). - initiated by a healthcare professional (for example, medical, nursing, social work, pharmacist) - have been delivered as the only heart failure disease management intervention - without home-visits or intensified clinic follow-up.   **Comparator:** Usual heart failure care,   - could involve standard post-discharge care - not any intensive attendance at cardiology clinics or clinic-based heart failure disease management program or home-visits. | - Remote monitoring of conditions other than heart failure - Studies with intensified clinic follow-up or any home visits which were conducted with the purpose of delivering education or performing clinical assessment. |
| Jones et al.  (2002)  [62] | People with problems commonly observed in persons age 65 and above | A nursing intervention using interactive technology targeting problems commonly observed in persons age 65 and above |  |
| Barlow et al.  (2007)  [63] | Frail elderly people or adults of any age with long-term conditions | - Home-based telecare services - Telecare as a core component, not merely one intervention among many | - Reviews - purely descriptive studies - Studies which focused only on implementation issues or satisfaction - Studies which had insufficient methodological detail - Focused on residential homes, prisons or other institutions - Studies which focused on terminal conditions |
| Franek  (2012)  [64] | Patients with COPD | **Intervention group:**  Research Question 1:   - Frequent transmission of a patient’s physiological data collected at home and without a health care professional physically present to health care professionals for routine monitoring through the use of a communication technology - monitoring combined with a coordinated management and feedback system based on transmitted data - telemonitoring as a key component of the intervention (subjective determination)   Research Question 2:   - Scheduled or frequent contact between patient and a health care professional via telephone or videoconferencing technology in the absence of transmission of patient physiological data - Monitoring combined with a coordinated management and feedback system based on transmitted data - Telephone support as a key component of the intervention (subjective determination)   **Comparator:**  Usual care as provided by the usual care provider for the control group | - Published in a language other than English - Intervention group (and not control) receiving some form of home visits by a medical professional, typically a nurse (i.e., telenursing) beyond initial technology set-up and education, to collect physiological data, or to somehow manage or treat the patient - Not recording patient or health system outcomes (e.g., technical reports testing accuracy, reliability or other development-related outcomes of a device, acceptability/feasibility studies, etc.) - Not using an independent control group that received usual care (e.g., studies employing historical or periodic controls) |
| van den Berg et al.  (2012)  [70] | Patients aged 60 or older | Interventions which were conducted directly in the homes of the patients, requiring a telemedical connection between a medical provider (e.g., hospital, private practice) and the patient in his home, addressing a defined diagnosis or medical indication | - Telemedicine concepts between medical providers only or between provider and patient outside his home (e.g., patients have to come to a primary care-practice to initiate a videoconference with a clinic) - Telemedicine concepts without the participation of medical professionals (e.g., computer-aided expert systems, web portals) - Studies including only healthy participants (e.g., for primary prevention purposes, preclinical studies) - Concepts targeted to primary prevention, convenience, or wellness |
| Foster et al.  (2014)  [71] | - Older adult population   (Older than 65 years  with a chronic illness) | A telehealth device that transmitted physiological or symptom perception data | - Study titles and/or abstracts that did not include any of the search terms - Systematic reviews - Grey literature (i.e., conference papers) - Studies conducted with older adults who have mental illness, cognitive impairment, or live in a nursing home |
| Nordheim et al.  (2014)  [72] | - Patients with arterial, venous and/or diabetes-related foot and/or leg ulcers participated. - Other types of ulcers if the authors provided separate results for the various types of ulcers. | **Intervention group:**  Transfer of digital still pictures or video consultation  **Comparator:**  Traditional face-to-face follow-up care (hereafter referred to as traditional follow-up) |  |
| Peretz et al.  (2016)  [73] | - Seniors with an average age greater than or equal to 65 years - Having at least one chronic condition | RPM^f^ services solely or in combination with other home healthcare services | - Any studies that used Markov or other cost simulation models - Any studies that did not separately report the costs of RPM^f^ equipment. |
| Frost et al.  (2017)  [74] | - Community-dwelling older people (mean age > =65) with mild frailty identified through a validated frailty scale which contained an intermediate classification between frail and robust (e.g., pre-frailty on the Fried phenotype, mild frailty on the Rockwood Clinical Frailty Scale, the modified physical performance test, the Electronic Frailty Index) - Studies reporting subgroup analyzes for a mildly frail subsample | **Intervention group:**  home- or community-based health promotion interventions (i.e. interventions that enable people to improve or increase control over their health  **Comparator:**  any comparison group | - Populations without a clear or validated definition of pre-frailty (e.g., ‘functionally limited’), pre-frail populations within a restricted range of health conditions and hospital or long-term care home populations to increase generalizability to the wider population - Inpatient interventions - Studies using biological markers only as outcomes (e.g., markers of inflammation, muscle composition) as single biomarkers are considered inadequate predictors of frailty - Other study designs (e.g., cohort, qualitative, non-randomized trials). - Studies combining an early frailty population with a frail or robust sample for analysis - Papers that reported the presence of a pre-frail group but did not report outcomes for this group (no response) |
| Karlsen et al.  (2017)  [75] | - Persons aged 60 years and over living in their own home - Living alone or with others (e.g., partners, children) - No limitations on disease type or chronic conditions. | Telecare as part of the home care services  Technological devices:   - “active systems” such as pendant alarms - “passive systems” as environmental sensors such as bed sensors, fall detectors, smoke alarms, stove temperature sensors and wandering detection sensors. | - Assistive devices (e.g., hearing aids, electric lift chairs), entertainment appliances (e.g., television, camera), and home automation/appliances (e.g., intercom systems, microwave ovens) - Exchange of information solely between professionals for diagnoses or referrals - Papers where potential users were asked about their attitudes or perceptions towards the technology - Usability studies in which developing innovative technology was the focus |
| Narasimha et al.  (2017)  [76] | Geriatrics | A direct communication between the care provider and the patient. | - Studies before 2000 or after Jul 2016 - Studies using such communication devices as EMR^e^ and self-measurement of vitals and medication adherence - Studies on peripheral medical devices used as part of telemedicine and general health monitoring using web-based applications, which did not consist of care-provider and patient communications - Virtual reality-based health training applications, literature reviews, posters, and extended abstracts - Articles discussing problems of the geriatric population, but not including results from a user research study |
| Gentry et al.  (2018)  [77] | Geriatric populations (primarily patients over age 50 or long term care population) | Intervention with alive, synchronous videoconferencing format | - Case reports - Descriptive reports without data - Poster presentations and conference abstracts Studies that did not provide diagnostic evaluation or treatment modality |
| Marx et al.  (2018)  [65] | - Older adult samples - Mean age of ≥65 years living independently in their own homes (including post-hospital discharge and outpatients) | - Intervention for managing risk or progression of protein-energy malnutrition - Community-based interventions delivered with at least 50% of the intervention contacts (frequency or duration) from telehealth methods - At least two points-of-contact made via telehealth - If an intervention was multidisciplinary and focused on more than just nutrition (e.g., support for dementia or stroke patients): there were at least two malnutrition-specific telehealth contacts within the larger intervention program - The telehealth intervention was given directly to the patient or to their family carer - Telehealth was considered as:   1) a synchronistic consultation with a health professional with point-of-contact via any telephone or internet-based method, or  2) an asynchronistic telephone- or internet-based intervention system. | - Participants in residential aged care or assisted living facilities - Studies where the intervention was delivered in both inpatient and community settings (e.g., during admission and then follow-up post-discharge) only if the intervention delivered in the community setting was of equal or greater duration than that delivered in the inpatient setting - Abstracts - Observational studies - Conference papers - Qualitative studies - Study protocols - Opinions - Commentaries - Review papers |
| Santana et al.  (2018)  [66] | Elderly Patients with Alzheimer's and their caregivers | **Intervention group:**  Telecare by nurses  **Comparator:**  Conventional follow-up | - Case reports, case series and expert opinion - Telecare that is used for medical diagnosis and without nursing performance - Theses and dissertations - Unfinished searches - Articles without determination of a clear methodology or that did not deal with the proposed theme |
| Batsis et al.  (2019)  [67] | - A mean participant age of 65 years and corresponding one standard deviation or range required to exceed 60 years - With any co-morbid physical and mental health conditions | - Ambulatory TMed^a^ care delivered either in-home, or in an assisted living or long-term care setting on the receiving end of the intervention (not acute or hospital settings) - Patient care with a health care provider or trained staff (i.e., physician, associate provider [advanced practice registered nurse or physician’s assistant], physical/occupational therapist, psychologists, social workers, or dietitians, etc.) on one end, and a patient on the receiving end - Peer-to-peer therapy for medical conditions, as it ultimately resulted in delivering patient care. | - TMed^a^ (remote monitoring, e-consultations or store-and-forward) related to remote medical education - Studies involving social media (i.e., Facebook or Twitter) |
| Christensen et al.  (2019)  [68] | People with unipolar depression aged 60+ years | VC^b^ by an outpatient tele-mental health service | - Only focused on non-video telephone intervention - Focused on self-administered interventions - Focused on video communication between providers |
| Costanzo et al.  (2019)  [69] | Subjects with a diagnosis of MCI^c^ or AD^d^ | The use of mobile, online, or other technologies, including any tele‐device such as phones and smartphones, computers and monitors, internet, wearable devices, and other new technologies, such as tablets, remotely used (including home care) to facilitate communication, consultation, and support | - Any other subtype of dementia (e.g., vascular dementia) - Technological tools used in the hospitals or for transferring Rx or MRI^e^ images - Conference abstracts, gray literature, commentary, and protocols |
| Aquilanti et al.  (2020)  [78] | - Elderly people requiring oral health care in nursing homes, in communities, or with in-home assisted - Of any sex, ethnicity, socio-economic status, and comorbidities | **Intervention group:**  Any type of oral health provision through the use of health information technology (screening, diagnosis, support, consultation, education, and any other kind of application in dental medicine)  **Comparator:**  The traditional face-to-face dental visits | - Reviews - Editorials - Commentaries - Letters - Book chapters - Reports on prospective ideas and futuristic scenarios (protocols included) - Dissertations |
| Kruse et al.  (2020)  [79] | Older adults (older than 50 years) | Some form of telehealth (including mHealth, eHealth, and all forms of telehealth) |  |
| Sekhon et al.  (2021)  [82] | Elderly individual, including rural population with dementia |  | - Studies that did not include elderly individuals - Telehealth not an outcome/intervention - Sample population did not include dementia patients - Did not include rural population - Article not accessible - No empirical data and/or not an original study |
| Elbaz et al.  (2021)  [83] | - Older adults aged 60 and above   With dementia | Telemedicine as the primary focus based in the COVID-era | - Did not include older adults (60+) - Mental health outcomes were not clearly described - The study was not conducted with individuals with dementia - No original data - Telemedicine was not the primary focus of the study - Article was not based in the COVID-era |
| Tam et al.  (2022)  [84] | - Mean age of participants was 60 years or above - Diagnosed with hypertension | **Intervention group:**  Text messaging used as a component  **Comparator:**  Employed usual care or RHE^h^ related to the use of medication and lifestyle modifications | - Kidney disease-related hypertension - Pulmonary hypertension - Effect of medication or supplement - Study protocols or unpublished theses |
| Markert et al.  (2021)  [85] | Older adults (aged 65 and older) | Health coaching (health or wellness coaching) combined with remote monitoring | - Older populations (aged 65 and over) were not included - Study that did not include remote monitoring (RPM^f^ or RAM^i^) - Study that did not include some form of coaching intervention |
| Wong et al.  (2022)  [86] | - Adults aged 60 or over - Living independently in the community | **Intervention group:**  Telehealth (defined as the use of apps, websites, WhatsApp, SMS text messages, email, social media such as Facebook or Twitter, telephone calls, tablets, software such as Zoom or Microsoft Teams, home remote monitoring devices [reactive or proactive], or any combination of these as a health care delivery channel)  **Comparator:**  A face-to-face or on-site care service | - Studies focusing on cognitively or functionally impaired older adults unable to perform self-care - Studies comparing 1 or more telecare interventions without a comparison with a control group or with a no intervention control group. |
| Rush et al.  (2022)  [87] | Older adult populations (aged 55+ years, to capture the vague and variable reporting of age categories) | Telehealth with rural or remote focus | - Studies which are not original research - Protocols - Reviews |
| Haimi et al.  (2022)  [88] | - The elderly population (age 65 years and more) - During the COVID-19 | Any sorts of use of telehealth tools in all aspects and levels of healthcare (primary, secondary, or tertiary), aimed for providing   - clinical and consultation services - assessment of symptoms - diagnosis - triage of patients, - either concerning Covid-19 symptoms or not | - Studies reporting methods which are not based on telemedicine technology - Duplicate publications - Review or opinion articles - Letters to the editor - Studies with incomplete information |
| Pool et al.  (2022)  [89] | Adults aged 60 years or above | Telehealth (e.g., telemedicine, telemonitoring, teleconsultation) in aged care services (provided to older persons, including but not limited to allied health care, primary care, and personal care) | Articles that did not meet the inclusion criteria, for example:   - Non-empirical articles (e.g., review, perspective) - Articles only focusing on EHR^j^, e-health, ICT^k^, exercise/fitness apps, wearable/mobile devices for self-monitoring - Studies that solely focus on technical aspects (e.g., algorithms, system architecture, wireless networks, sensor design, authentication scheme) and neglected to study the humanistic aspects of using telehealth (e.g., user experience, attitude, behavior) - Studies that were not related to adults aged 60 years or above |
| Al-Naher et al.  (2022)  [80] | - Patients diagnosed with adult chronic heart failure of all severities   OR   - Their carers or the health care professionals involved in their care. | Remote care programs:   - Defined as any intervention accessible from the patient’s home or local community - Provides the patient with education, assessment, investigation results   OR  Otherwise replaces a service that would normally be offered within a formal clinical setting | - Nonprimary studies - Studies with no qualitative element in the collection or analysis of data - Studies that did not include patients with adult chronic heart failure and neither their carers nor health care professionals - Non-English studies - Interventions already established for heart failure care within national/international guidelines - Interventions involving implantable devices where user engagement is not a factor |
| Murphy et al.  (2020)  [81] | All patients who were referred for a Geriatrician led outpatient assessment | **Intervention:** those who received an outpatient assessment via a virtual geriatric clinic consultation  **Within study comparator group:** those which did not utilize a virtual clinic consultation. | - Model of care was not an outpatient clinic setting and it did not involve specialist Geriatric physician consultation (i.e. studies which reported on telemedicine from rural hospitals to tertiary assessments) - Studies involving emergency assessments of patients by telemedicine (as this would be out of the remit of a standard geriatric outpatient visit) |

*^a^TMed: Telemedicine, ^b^VC: Videoconferencing, ^c^MCI: Mild Cognitive Impairment, ^d^AD: Alzheimer's Disease, ^d^MRI: Magnetic Resonance Imaging, ^f^RPM: Remote Patient Monitoring, ^g^EMR: Electronic Medical Records, ^h^RHE: Routine Health Education, ^i^RAM: Remote Activity Monitoring, ^j^EHR: Electronic Health Records, ^k^ICT: Information and Communication Technologies*
