# Supplementary material for: Domains, Feasibility, Effectiveness, Cost, and Acceptability of Telehealth in Aging Care: Scoping Review of Systematic Reviews
Source: JMIR Aging. 2023 Apr 18;6:e40460. doi: 10.2196/40460 (PMC10155091; doi:10.2196/40460)
Supplement: Multimedia Appendix 5 [file aging_v6i1e40460_app5.docx]

Multimedia Appendix 5. Summary of the effectiveness outcomes of telehealth

| Individual-level Outcomes | | | |
| --- | --- | --- | --- |
| REFERENCES | **CLINICAL BENEFITS FINDINGS** | **HEALTH LITERACY & KNOWLEDGE FINDINGS** | **BEHAVIORAL OUTCOMES** |
| Inglis et al.  (2015)  [29] | - Compared to usual care, remotely monitoring older patients with heart failure using structured telephone support or telemonitoring reduces mortality rates. - Structured telephone support also reduces the risk of heart failure-related hospitalizations but there was insufficient evidence to conduct a meta-analysis of the effect of telemonitoring. - In the primary meta-analysis reported in the published Cochrane Review, a modest yet statistically significant reduction in all-cause hospitalization was observed. The protective effect of remote monitoring on all-cause hospitalizations was not sustained when only studies that enrolled a majority of older people were examined. |  |  |
| Jones et al.  (2002)  [62] | - A small but persuasive set of research projects identified the important components of home care that could be delivered via Telehealth applications, illustrated the feasibility and potential health benefits of Telehealth innovations designed to intervene in significant health problems of elders. - The findings from the studies demonstrate the effectiveness of Telehealth assessment strategies for complex and meaningful patient problems. Thus, the use of Telehealth approaches for clinical assessment, while not ready for wide-scale clinical deployment, shows great promise in assisting nurses in this most important component of the nursing process. |  |  |
| Barlow et al.  (2007)  [63] | **Vital signs monitoring:**  Evidence about the effects of telemonitoring on clinical outcomes was inconsistent. A large number of studies have been conducted, with about half suggesting clinical benefits and the other half finding no effect.  **Safety and security monitoring:**  There was insufficient rigorous evidence about the effects of alert systems such as fall detectors and community alarms, on either individual or system outcomes. **Information provision:**   - There was evidence that proactive support from practitioners by telephone can improve clinical outcomes. - Based on the evidence reviewed, the most effective telecare interventions appear to be automated vital signs monitoring (for reducing health service use) and telephone follow up by nurses (for improving clinical indicators and reducing health service use). However, it is important to note that just because there is insufficient evidence about some interventions, this does not mean that those interventions have no effect. |  |  |
| van den Berg et al.  (2012)  [70] | None of the included study reports a significantly better outcome for the control group. If this would be an indication of publication bias, then telecare would appear to be more beneficial than it is in reality. However, while this cannot completely be excluded, the many positive examples provided in the literature indicate the considerable potential of these new modalities. |  | A regular personal monitoring and individual supporting contact between healthcare professional or in form of telemedical measurements seems to have a positive influence on the adherence to instructions to behavioral changes and adherence. |
| Nordheim et al.  (2014)  [72] | The evidence that we identified still renders it inconclusive whether telemedicine management of people with diabetes-related foot ulcers may be an equivalent alternative to traditional follow-up concerning the healing time of the ulcers. |  |  |
| Frost et al.  (2017)  [74] | No statistical comparison for pre-frail group; slightly higher transitions to non-frail and frail in usual care. |  |  |
| Gentry et al.  (2018)  [77] | There are RCT's^a^ showing efficacy of these TMH^c^ interventions in improving depression, insomnia, and QoL^d^. |  |  |
| Marx et al.  (2018)  [65] | - Compared with usual care, this review found evidence that malnutrition-related interventions delivered via telehealth are effective in improving quality of life and protein-intake, although confidence in the estimated effect sizes for these outcomes is low to very low. - Pooled data did not find statistical significance in improvements in nutrition status, physical function, energy intake, falls, hospital readmissions and all-cause mortality. improvements in nutrition status, physical function, energy intake, falls, hospital readmissions and all-cause mortality. - Overall, this review found telehealth is an effective method to deliver malnutrition-related interventions to older adults living at home, and is likely to result in clinical improvements compared to usual care or no intervention. |  |  |
| Santana et al.  (2018)  [66] | No results were found to improve the functional capacity of the elderly people. | - They also describe a better understanding of basic pathology and comorbidities. - The use of telecare for elderly people with Alzheimer's and their caregivers with direct nurses' performance was able to improve the elderly's behavior management skills and the perception of the caregiver's overload. |  |
| Christensen et al.  (2019)  [68] | Four studies combined clinical outcome and satisfaction, and in the RCT^a^ studies presented, there was a correlation between clinical effect and satisfaction, which could indicate that the patients in VC^b^ groups also had a clinical effect. |  |  |
| Costanzo et al.  (2019)  [69] | - The results were promising, and, in particular, the interventions to support patients with possible orientation problems appear to be very useful in terms of prolonging patient's autonomy. - Psychosocial interventions-typically involving support groups, individual counselling, and education-have only modest therapeutic benefits, while telehealth care combined could reduce family caregiver burden, improve stress mastery, and family and social function, finally resulting in a global improvement of caregiver's quality of life. - The family therapy intervention by itself did not have a significant effect on depressive symptoms, but the use of technology probably enabled caregivers to receive additional individualized support and to facilitate the resolution of some family conflicts, enhancing the effect of eventual antidepressant. - Telemedicine seems to show a big potential in increasing the safety of these patients by preventing wandering behaviors and falls in stimulating their cognitive functions, in prolonging their autonomy, and, ultimately, in releasing the burden from their caregivers' shoulders. | Thus, although the studies were various, all came to the conclusion that patients with cognitive deficits can relearn everyday skills through the use of different technological tools, in particular, when learning methods with error reduction are used. |  |
| Aquilanti et al.  (2020)  [78] | most of papers stated that Teledentistry is comparable to or even better than the conventional alternative, but conclusive statements are not possible to be drawn and publication bias could be met. Only few studies are well constructed and reported a controlled comparison between Teledentistry application and face-to-face alternatives. |  |  |
| Kruse et al.  (2020)  [79] | The research supports strong medical outcomes incident to the use of telehealth as follows: decreased psychological distress, increased autonomy, increased cognitive ability, and many others. This review supports an increased quality of life for those who adopt telehealth. |  |  |
| Tam et al.  (2022)  [84] | Results of the meta-analysis showed that text messages delivered every 7–12 days could significantly reduce SBP^e^ and DBP^f^. |  | The integration of TMIs^g^ could have a moderate effect on improving medication adherence. |
| Wong et al.  (2022)  [86] | Overall, the findings of this review suggest that nurse-led telehealth programs may improve the QoL^d^, self-efficacy, and depression levels of community-dwelling older adults when compared with the usual face-to-face care. However, no significant differences across groups were noted in hospital admissions. |  |  |
| Rush et al.  (2022)  [87] | Telehealth was found to be useful for promoting health outcomes among rural older adults across several diverse contexts. This broad usefulness of telehealth presents a possible solution to the poor mental health (e.g., suicide), unhealthy behaviors (e.g., smoking), obesity, and chronic diseases (e.g., cardiovascular disease, diabetes) that are higher in rural and remote areas than urban areas. |  | This broad usefulness of telehealth presents a possible solution to the poor mental health (e.g., suicide), unhealthy behaviors (e.g., smoking), obesity, and chronic diseases (e.g., cardiovascular disease, diabetes) that are higher in rural and remote areas than urban areas. |
| Murphy et al.  (2020)  [81] | This review has demonstrated the potential benefits to patients with a virtual geriatric clinic model of care, including successful polypharmacy reviews, reductions in acute hospitalization events. |  |  |

| System-level Outcomes | | |
| --- | --- | --- |
| References | **EFFICACY** | **HEALTHCARE SYSTEM USE** |
| Jones et al.  (2002)  [62] | A small but persuasive set of research projects identified the important components of home care that could be delivered via Telehealth applications, demonstrated the equivalence of technology-mediated assessment with face-to-face approaches |  |
| Barlow et al.  (2007)  [63] | **Safety and security monitoring:** There was insufficient rigorous evidence about the effects of alert systems such as fall detectors and community alarms, on either individual or system outcomes. | **Vital signs monitoring:** There was evidence that for people with certain conditions, monitoring can help to reduce health service use, including reducing hospital admissions and costs.  **Information provision:** Telephone follow-up after hospital discharge was also associated with reduced health service use. There was inconsistent evidence about the effects of education and support provided via email and the Internet.  Based on the evidence reviewed, the most effective telecare interventions appear to be automated vital signs monitoring (for reducing health service use) and telephone follow up by nurses (for improving clinical indicators and reducing health service use). However, it is important to note that just because there is insufficient evidence about some interventions, this does not mean that those interventions have no effect. |
| Franek  (2012)  [64] | The economic impact of home telemonitoring is uncertain and requires further study. | There is a trend towards significant increase in time free of hospitalization and use of other health care services with home telemonitoring, but these findings need to be confirmed further in randomized trials of high quality.  Low quality evidence finds significant benefit in favor of telephone-only support for self-efficacy and emergency department visits when compared to usual care, but non-significant results for hospitalizations and hospital length of stay. |
| van den Berg et al.  (2012)  [70] | None of the studies report about a transfer of the telemedical concept into regular healthcare. The lack of transfer, and concomitant with this, a reliable reimbursement of the provided services, is among the major problems in the establishment of successful telemedicine concepts in the healthcare system. |  |
| Gentry et al.  (2018)  [77] | There is emerging evidence that memory disorder clinics provided via telemedicine are feasible, well accepted and able to produce comparable outcomes to IP^h^ care. However, these studies are of low methodologic quality, and randomized controlled trials are needed. |  |
| Marx et al.  (2018)  [65] | Although the clinical- and cost-efficacy of telehealth interventions compared with home visits are not established, compared with no intervention, telehealth interventions are cost-effective and have increased feasibility from a health care point of view making it more likely patients will receive the intervention |  |
| Santana et al.  (2018)  [66] | There was a direct action of the nurse in the distance monitoring and positive return by the caregivers. Thus, it is estimated that distance monitoring through the use of technologies, especially in cases of long-term care and dependency, tends to increase as in the case of the person with Alzheimer's and their caregivers. |  |
| Christensen et al.  (2019)  [68] |  | Video consultations seemed especially useful in order to improve access to health services in remote areas and make equal access to treatment possible. |
| Costanzo et al.  (2019)  [69] | The most used diagnostic technology in the articles we reviewed was video conferencing and video monitoring. Half of the studies compared the efficacy of IP^h^ diagnosis with the efficacy of diagnosis made through a TM^i^, usually via video conference, and they found there is no difference in the efficacy of the two methods. While the authors concluded that further studies are needed, primarily to improve the accuracy of the results, they also agreed that new technologies are a promising new tool for the early detection of clinically relevant changes in MCI^j^ and AD^k^ patients, leading to earlier therapeutic intervention and ultimately slowing down the progression of the disease. |  |
| Sekhon et al.  (2021)  [82] | Adherence and cognitive tests reported mixed results regarding the reliability of telemedicine, due to (1) the testing conditions and (2) the accessibility of telemedicine, which yield inconclusive results as to whether telemedicine can improve the management of dementia in rural geriatric individuals. |  |
| Elbaz et al.  (2021)  [83] |  | Nevertheless, a consensus remains that telemedicine could positively impact patients and their access to healthcare |
| Markert et al.  (2021)  [85] | - Health coaching that incorporates telehealth technologies has been implemented in older populations with mixed results. As much as 10 of the 13 studies reviewed found this method of health coaching to provide effective outcomes. - These results indicate that coaching effectiveness may not be dependent on the method of communication with the patient. - One of the more prominent findings identified in this review was the dependence on a human to provide health coaching and interaction with the patient. Thus, the outcomes were probably heavily reliant on a human in the process. |  |
| Wong et al.  (2022)  [86] |  | Taken together, the findings seem to suggest that well-designed nurse-led services delivered by trained staff may be a promising program of care that can complement and extend existing services from the health care facility to the home/community. |
| Haimi et al.  (2022)  [88] |  | Although older patients may benefit the most from using home telehealth visits, which improve their access to care, especially in the Covid-19 era, in which the necessity of such solutions was undoubtedly proved, still, paradoxically, there are not enough telehealth solutions addressed and aimed at this special population, and it seems that not enough efforts were made to satisfy this purpose. |
| Murphy et al.  (2020)  [81] | - Cognitive impairment and polypharmacy were amenable to assessment with the virtual geriatric clinic model in the included studies, but other important geriatric syndromes such as frailty, sarcopenia were not evaluated. - While there is evidence of productivity there is a need for solid collaboration from referring clinicians. A theme across the included studies was the need for strong partnership. Success was attributed to strong links between departments | This review has demonstrated the potential benefits to patients with a virtual geriatric clinic model of care, including successful polypharmacy reviews, reductions in acute hospitalization events, and shortened waiting times for patients for first review. |

^a^RCT: Randomized Controlled Trial, ^b^VC: Videoconferencing, ^c^TMH: Telemental Health, ^d^QoL: Quality of Life, ^e^SBP: Systolic Blood Pressure, ^f^DBP: Diastolic Blood Pressure, ^g^TMI: Telemedicine Intervention, ^h^IP: In-Person, ^i^TM: Telemedicine, , ^j^MCI: Mild Cognitive Impairment, ^k^AD: Alzheimer’s Disease
