# Supplementary material for: Domains, Feasibility, Effectiveness, Cost, and Acceptability of Telehealth in Aging Care: Scoping Review of Systematic Reviews
Source: JMIR Aging. 2023 Apr 18;6:e40460. doi: 10.2196/40460 (PMC10155091; doi:10.2196/40460)
Supplement: Multimedia Appendix 6 [file aging_v6i1e40460_app6.docx]

Multimedia Appendix 6. Summary of the feasibility outcomes of telehealth

| REFERENCES | ADHERENCE TO THE INTERVENTION |
| --- | --- |
| Marx et al.  (2018)  [65] | Seven of the nine intervention studies used telephone consultations, which overall had much lower attrition rates (0-31%) than those which used telemonitoring devices (50-61%). |
| Sekhon et al.  (2021)  [82] | There were mixed results on the actual rate of adherence to telehealth and on the implementation of telemedicine specialists’ recommendations |
| Rush et al.  (2022)  [87] | Usage findings were mixed with half of the studies reporting low adherence and modest attrition rates. A number of well-known factors, such as technology failure or not achieving behavioral goals, contributed to low adherence, a unique finding of this review was the variability in adherence rates according to the nature of the telehealth interventions. |
