# Supplementary material for: Domains, Feasibility, Effectiveness, Cost, and Acceptability of Telehealth in Aging Care: Scoping Review of Systematic Reviews
Source: JMIR Aging. 2023 Apr 18;6:e40460. doi: 10.2196/40460 (PMC10155091; doi:10.2196/40460)
Supplement: Multimedia Appendix 7 [file aging_v6i1e40460_app7.docx]

Multimedia Appendix 7. Summary of the cost-benefit outcomes of telehealth

| References | Cost-benefit outcomes |
| --- | --- |
| Peretz et al.  (2016)  [73] | - RPM^a^ programs monitoring a single vital sign were likely to be less costly than those covering multiple. Programs monitoring one condition (CHF^b^ or HTN^c^) seemed less costly than those covering respiratory diseases or multiple conditions and that programs implemented in North America seemed more costly than those implemented in Europe or New Zealand. Though only one of these findings is statistically significant, one can hypothesize that each of these individual characteristics could potentially contribute to the heterogeneous results obtained, making it all the more important to understand how these factors contribute to cost differences across studies. - In addition, the statistically significant finding that indicates that programs which cover CHF^b^ or HTN^c^ are less costly than those which cover respiratory diseases or multiple conditions can be explained by two factors: patient complexity and number of vital signs being monitored. - Lastly, geography seems to have an influence on cost, with North American RPM^a^ programs being more expensive when compared to other regions, which does not seem logical and is likely due to other confounding variables. - No reliable cost estimates for the implementation of RPM^a^ programs are available in the literature. On the basis of cost, this review suggests that RPM^a^ programs are more suitable for certain chronic conditions and grouping similar characteristics of an RPM^a^ program, such as the type of condition, the number of vital signs monitored, etc. would yield more useful, comparable data that is generalizable and could serve to inform policy and decision makers. |
| Gentry et al.  (2018)  [77] | - There is limited data on cost-effectiveness of TMH^d^ in the elderly. - Although these studies provide support for the cost-benefit to patients and caregivers, the cost-effectiveness for health-care organizations has not been shown. - The one available study on cost-effectiveness found that increasing health care costs did not differ between VC^e^ and IP^f^ treatment. |
| Marx et al.  (2018)  [65] | Compared with no intervention, telehealth interventions are cost-effective and have increased feasibility from a health care point of view making it more likely patients will receive the intervention |
| Aquilanti et al.  (2020)  [78] | When comparing Teledentistry to face-to-face oral examinations, a reduction in costs was likely to be detected |
| Murphy et al.  (2020)  [81] | Virtual geriatric clinics appear to be cost effective. Exact cost per visit was provided in just two studies but it is interesting to note that there were significant discrepancies within the different costing models used, resulting in an almost sixfold difference in savings, depending on the cost model used. A clear breakdown of cost calculations is important to be able to make valid comparisons between studies, with clarity over re-imbursement claims crucial in the expansion of virtual clinics. |
| Rush et al.  (2022)  [87] | Evidence from this review points to direct cost savings for health care system and/or rural older adults with the use of telehealth and is strengthened by the fact that the few studies that examined cost-effectiveness were all medium to high quality, and all but one had a low risk of bias. Although cost savings related to hospital and inpatient services were observed, what was not often addressed were costs to patients. Many of the cost savings accrued to the patient were because of reduced travel costs, which could remove significant financial burdens for rural patients. |

^a^RPM: Remote Patient Monitoring, ^b^CHF: Congestive Heart Failure, ^c^HTN: Hypertension, , ^d^TMH: Telemental Health, ^e^VC: Videoconferencing, ^f^IP: In-Person
