# Supplementary material for: Domains, Feasibility, Effectiveness, Cost, and Acceptability of Telehealth in Aging Care: Scoping Review of Systematic Reviews
Source: JMIR Aging. 2023 Apr 18;6:e40460. doi: 10.2196/40460 (PMC10155091; doi:10.2196/40460)
Supplement: Multimedia Appendix 8 [file aging_v6i1e40460_app8.docx]

Multimedia Appendix 8. Summary of the acceptance outcomes with factors affecting telehealth utilization

| REFERENCES | SATISFACTION, ACCEPTABILITY, ATTITUDE, EXPERIENCE, USABILITY | FACILITATORS AND BARRIERS OF USING THE TELEHEALTH INTERVENTIONS |
| --- | --- | --- |
| Inglis et al.  (2015)  [29] | - The lack of recruitment of elderly patients into trials evaluating the effectiveness of interventions for heart failure is an alarming finding, given the fact that heart failure becomes more prevalent as age is increased. - The current accumulated evidence supports the use of structured telephone support and telemonitoring in elderly heart failure patients and goes some way towards dispelling a popularly held belief among some clinicians that elderly patients will not benefit from telehealth interventions. |  |
| Jones et al.  (2002)  [62] | A small but persuasive set of research projects identified the important components of home care that could be delivered via Telehealth applications, depicted the somewhat neutral to positive reactions held by elders and nurses to Telehealth applications. |  |
| van den Berg et al.  (2012)  [70] |  | In some studies it was reported that patients dropped out because they refused the technical installation and use of telemedical devices. One study including patients with Alzheimer’s disease, emphasized the importance of the cooperation of caregiving relatives. These “marginal notes” indicate possible problems of some patients in handling the telemedicine intervention. |
| Foster et al.  (2014)  [71] |  | **Facilitators to the acceptance of telehealth by older adults:**  (1) devices that use fewer buttons,  (2) automatic transmission of information,  (3) utilizing low-tech platforms (i.e., telephone, TV),  (4) devices that generate reminders or alerts,  (5) providing both visual and audio guidance,  (6) user-friendly images appropriate for the elderly.  **Barriers to the use of telehealth technologies:**  (1) font size, unusual characters (difficult to read);  (2) bland graphics and poor color contrast;  (3) using devices with widgets (older patients lack poor fine motor eye-hand coordination);  (4) use of a computer mouse (difficult to use with arthritic hands);  (5) unskilled on the use of a smartphone or a computer;  (6) multiple screen transitions to complete a task;  (7) menu bars that contain several layers; and  (8) inappropriate size of a smartphone (too big or too small; frail patients who have diminished grip strength may have problems handling the device).  For older adults who are not accustomed to using technology, telehealth may also represent a cultural change, something that has to be kept in mind when adopting telehealth.   Delay in responses, lack of feedback, and technical problems can all lead to frustration and reduce motivation for patients to continue self-care monitoring activities. |
| Karlsen et al.  (2017)  [75] | The categories generated seven synthesized findings on community-dwelling older adults experience:   1) Aging in place is desired; however, it may also be related to feeling isolated and lonely.  2) Telecare contributes to safety, security, and aging in place.  3) Privacy is not seen as a problem by most older adults because the technology is intended to help them live safely in their own home.  4) Some telecare devices have side effects, especially new technology. Some devices do not work outside the home, thus limiting active aging.  5) Some older adults experience a misfit between technology and needs. They must see the value of a telecare device to use it.  6) Telecare may enforce an identity with negative connotations on older adults, as frail and helpless people. Autonomy is considered important.  7) Lack of understanding can hamper the correct use of telecare. |  |
| Narasimha et al.  (2017)  [76] | 65% of the geriatric population wants to keep abreast of current advancements. The majority of studies included in this systematic review were conducted late in the telemedicine system design cycle, either at the end of the system design or after implementing the system. | - In telemedicine systems, these visual problems may manifest in older patients experiencing difficulty reading the buttons on a phone or laptop. - It was found in the studies reviewed here that patients with hearing disabilities found it difficult to communicate using telemedicine equipment, and participants also reported interference between a videophone and the hearing aid in the study. - An effective device or display screen is one that helps users easily navigate through a series of activities to achieve a final goal. The success of this process depends on cognitive ability, participants also reported interference between a videophone and the hearing aid in the study. which, in older adults may be affected as a result of age, resulting in reduced working memory capacity, perceptual speed, reasoning ability, and attention. - A loss in motor skills and motor coordination in older adults also affects their ability to carry out tasks. |
| Gentry et al.  (2018)  [77] | Patients reported high satisfaction with these TMH^d^ treatments. | - Low bandwidth, hearing or vision difficulties, and delirium in patients can present difficulty in the accuracy of some assessments. - Barriers to telemedicine can be divided into broad categories such as the quality/cost of technology, clinical factors (patients and providers), regulatory (state law, licensure), legal (liability, malpractice, privacy, safety), and reimbursement. |
| Marx et al.  (2018)  [65] | As no study reported on participant satisfaction, the reasonably low attrition rates in the telephone consultation groups imply that telehealth is a feasible and acceptable option in this patient group; however, asynchronistic approaches which rely on computerized devises may be less desirable as at least half of all participants were lost-to-follow-up. |  |
| Santana et al.  (2018)  [66] | Another satisfaction result found in the studies was the usability of the telecare system and the satisfaction with the access to the professionals without leaving home. |  |
| Batsis et al.  (2019)  [67] | Our findings demonstrate that TMed^a^ interventions are feasible and acceptable among older adults and that similar outcomes are achievable compared to usual, in-person care. | Providers are often hesitant in recommending technologies in older adults due to potential physical, sensory, cognitive and visual-spatial abnormalities. |
| Christensen et al.  (2019)  [68] | Most studies reported positive findings in regard to patients' and providers' experiences when using VCs^b^ and that the use of VCs^b^ is comparable to FTFT^c^. | - Concerns about the use of VCs^b^ for older people with possible sensory impairment and unfamiliarity with and resistance to the use of telepsychiatric equipment are voiced. When comparing studies in this review, technical challenges are also mentioned by patients and providers, especially in studies that include older patients. - One major barrier was reported in one study to be difficulty in obtaining a referral for VC^b^ treatment from family physicians, due to their scepticism regarding whether the patients would benefit from VC^b^ assessment. |
| Costanzo et al.  (2019)  [69] | - Younger caregivers seemed to be more comfortable with using the Internet and they experienced fewer access barriers to Internet use, being more motivated to use the service. - All of the authors agreed in recognizing the potential of telemedicine in supporting people with dementia and their caregivers. |  |
| Aquilanti et al.  (2020)  [78] | Overall, a high level of acceptability of Teledentistry was reported in the majority of the articles included in this review among patients, patients’ families, and caregivers. |  |
| Kruse et al.  (2020)  [79] |  | The most frequent barriers were lack of desire, cost, lack of technical support, visual acuity, social implications of use, ownership of technology, privacy and security, medical literacy, trust of the internet, mental acuity, hand-eye coordination, auditory acuity, and computer anxiety. Each of these barrier areas could present hurdles for elderly people dealing with telehealth and reasons to not use it. |
| Sekhon et al.  (2021)  [82] | - Overall, there was general satisfaction with telemedicine (for patients, caregivers, referring physicians and healthcare professionals) - Inspecting those variables yielded positive results for the vulnerable rural, elderly population. Generally, participants, families and health care professionals were satisfied with telemedicine, and found it to be convenient. | Gaining a referral to a specialist that utilizes telemedicine is also a barrier, even though all the rural physicians reported that they would use telemedicine in the future. |
| Elbaz et al.  (2021)  [83] | In all seven studies, the older adults showed a trend toward admiration for these technologies | The current systematic review revealed several barriers remaining within telemedicine practices applied to the geriatric population, specifically, individuals living with dementia, such as the inability to deal with technical issues, connectivity problems, as well as the loss of information due to being unable to properly examine the patient. |
| Rush et al.  (2022)  [87] | - Evidence was mixed as to the ease or difficulty rural older adults experienced in using telehealth, reflecting the impact of a number of factors consistent with other literature. - The evidence for intention to use telehealth or preferences as a proxy to use was limited, as few studies examined intention to use directly; however, the studies were medium to high quality with a low risk of bias. |  |
| Pool et al.  (2022)  [89] | - Privacy concerns have an impact on user attitudes intentions to adopt, and their actual use of telehealth. - Included studies in our review hinted at both the benefits and privacy risks that may arise from the use of telehealth. However, they generally did not measure the perceived benefits and risks of telehealth, and subsequent impacts on the adoption and use. | - Our results indicate that the older persons express more privacy concerns with the use of telehealth in smart home settings, where there is more surveillance and telemonitoring, e.g., capturing behavioral data and video recording. However, in the videoconferencing consultations, the privacy concerns are found to be less salient. Our review illustrates several examples of privacy concerns in video monitoring and home telehealth. - In the contexts of formal and informal care (care provided by family, relatives, or friends), telemonitoring in certain spaces such as bathrooms can raise privacy concerns. - Covering all locations in telehealth increases the safety and effectiveness of aged care service delivery. However, in telehealth service delivery other modes of telemonitoring should be available depending on contexts such as the health condition of users, emergency, non-emergency services, and more importantly older persons users’ preferences for controlling their data. - Ethical and legal issues arise once a violation of data regulations occurs in telehealth use. |
| Murphy et al.  (2020)  [81] | - Overall it is acknowledged that the patients were satisfied with the process, through various self-reported measures or Likert scales. - Satisfaction rates did not differ between studies which had an initial in person consultation and those without an initial in person consultation, indicating both are acceptable. |  |

^a^TMed: Telemedicine, ^b^VC: Video Conferencing, ^c^FTFT: Face-To-Face Treatment, ^d^TMH: Telemental Health
